# Current and Projected Mortality and Hospitalization Rates Associated With Conditional Cash Transfer, Social Pension, and Primary Health Care Programs in Brazil, 2000-2030

**DOI:** 10.1001/jamanetworkopen.2024.7519

**Published:** 2024-04-22

**Authors:** Temidayo James Aransiola, Daniella Cavalcanti, José Alejandro Ordoñez, Philipp Hessel, Ana L. Moncayo, Carlos Chivardi, Alberto Sironi, Renato Tasca, Tereza Campello, Rômulo Paes-Sousa, Gulnar Azevedo e Silva, Felipe Alves Rubio, Luis Eugenio de Souza, James Macinko, Davide Rasella

**Affiliations:** 1 Institute of Collective Health (ISC) at the Federal University of Bahia (UFBA), Salvador, Brazil; 2 Alberto Lleras Camargo School of Government at the Universidad de los Andes, Bogotá, Colombia; 3 Swiss Tropical & Public Health Institute, Basel, Switzerland; 4 Centro de Investigación para la Salud en América Latina (CISeAL), Pontificia Universidad Católica del Ecuador, Ecuador; 5 Health Research Consortium (CISIDAT), Cuernavaca, Mexico; 6 Center for Health Economics, University of York, York, United Kingdom; 7 Barcelona Institute for Global Health (ISGlobal), Hospital Clínic–Universitat de Barcelona, Barcelona, Spain.; 8 Institute of Studies for Health Policies, Rio de Janeiro, Brazil; 9 Center for Epidemiological Research in Nutrition and Health (NUPENS) at the University of São Paulo (USP), São Paulo, Brazil; 10 René Rachou Institute at Fiocruz, Belo Horizonte, Brazil; 11 Hesio Cordeiro Institute of Social Medicine at the State University of Rio de Janeiro, Rio de Janeiro, Brazil; 12 University of California, Los Angeles

## Abstract

**Importance:**

The health outcomes of increased poverty and inequalities in low- and middle-income countries (LMICs) have been substantially amplified as a consequence of converging multiple crises. Brazil has some of the world’s largest conditional cash transfer (Programa Bolsa Família [PBF]), social pension (Beneficio de Prestacão Continuada [BPC]), and primary health care (Estratégia de Saúde da Família [ESF]) programs that could act as mitigating interventions during the current polycrisis era of increasing poverty, slow or contracting economic growth, and conflicts.

**Objective:**

To evaluate the combined association of the Brazilian conditional cash transfer, social pension, and primary health care programs with the reduction of morbidity and mortality over the last 2 decades and forecast their potential mitigation of the current global polycrisis and beyond.

**Design, Setting, and Participants:**

This cohort study used a longitudinal ecological design with multivariable negative binomial regression models (adjusted for relevant socioeconomic, demographic, and health care variables) integrating the retrospective analysis from 2000 to 2019, with dynamic microsimulation models to forecast potential child mortality scenarios up to 2030. Participants included a cohort of 2548 Brazilian municipalities from 2004 to 2019, projected from 2020 to 2030. Data analysis was performed from September 2022 to February 2023.

**Exposure:**

PBF coverage of the target population (those who were poorest) was categorized into 4 levels: low (0%-29.9%), intermediate (30.0%-69.9%), high (70.0%-99.9%), and consolidated (≥100%). ESF coverage was categorized as null (0), low (0.1%-29.9%), intermediate (30.0%-69.9%), and consolidated (70.0%-100%). BPC coverage was categorized by terciles.

**Main outcomes and measures:**

Age-standardized, all-cause mortality and hospitalization rates calculated for the entire population and by age group (<5 years, 5-29 years, 30-69 years, and ≥70 years).

**Results:**

Among the 2548 Brazilian municipalities studied from 2004 to 2019, the mean (SD) age-standardized mortality rate decreased by 16.64% (from 6.73 [1.14] to 5.61 [0.94] deaths per 1000 population). Consolidated coverages of social welfare programs studied were all associated with reductions in overall mortality rates (PBF: rate ratio [RR], 0.95 [95% CI, 0.94-0.96]; ESF: RR, 0.93 [95% CI, 0.93-0.94]; BPC: RR, 0.91 [95% CI, 0.91-0.92]), having all together prevented an estimated 1 462 626 (95% CI, 1 332 128-1 596 924) deaths over the period 2004 to 2019. The results were higher on mortality for the group younger than age 5 years (PBF: RR, 0.87 [95% CI, 0.85-0.90]; ESF: RR, 0.89 [95% CI, 0.87-0.93]; BPC: RR, 0.84 [95% CI, 0.82-0.86]), on mortality for the group aged 70 years and older, and on hospitalizations. Considering a shorter scenario of economic crisis, a mitigation strategy that will increase the coverage of PBF, BPC, and ESF to proportionally cover the newly poor and at-risk individuals was projected to avert 1 305 359 (95% CI, 1 163 659-1 449 256) deaths and 6 593 224 (95% CI, 5 534 591-7 651 327) hospitalizations up to 2030, compared with fiscal austerity scenarios that would reduce the coverage of these interventions.

**Conclusions and relevance:**

This cohort study’s results suggest that combined expansion of conditional cash transfers, social pensions, and primary health care should be considered a viable strategy to mitigate the adverse health outcomes of the current global polycrisis in LMICs, whereas the implementation of fiscal austerity measures could result in large numbers of preventable deaths.

## Key Points

**Question:**

Are conditional cash transfers, social pensions, and primary health care programs associated with a mitigation of the health consequences of the polycrisis in low- and middle-income countries (LMICs)?

**Findings:**

This cohort study evaluated 2548 Brazilian municipalities from 2004 to 2019, integrating forecasting models up to 2030. Consolidated conditional cash transfers, social pensions, and primary health care coverages were associated with reductions of up to 8% in overall mortality rates; and the expansion of these programs were projected to prevent 1 305 359 deaths by 2030.

**Meaning:**

These results suggest that the expansion of conditional cash transfers, social pensions, and primary health care programs could be considered an effective strategy to mitigate the adverse health outcomes of the current polycrisis in LMICs.

## Teaser

This cohort study investigates the Brazilian conditional cash transfer, social pension, and primary health care programs and the reduction of morbidity and mortality from 2000 to 2019 and forecasts these programs’ potential mitigation of the current global polycrisis and beyond.

## Introduction

In low- and middle-income countries (LMICs), the development of welfare state programs during the last decades has been substantially reflected in the expansion of social protection policies, mainly based on conditional cash transfers, social pensions, and universal health coverage focused on the expansion of primary health care.^1^ While welfare state programs substantially contributed to the reduction of mortality in LMICs during periods of social and economic growth, they have also been able to mitigate negative effects on health during economic recessions.^2^ One of the main consequences of the COVID-19 pandemic has been the acute increase in poverty and social inequalities worldwide. More than 120 million people have already been pushed back into extreme poverty, with a global poverty rate projected to reach 7% by 2030.^3^ Moreover, the global economic consequences of the recent war in Ukraine are expected to push 71 million more people into severe poverty.^4^ The current worldwide surging inflation and debt tightening are also contributing to worrisome economic scenarios, with a forecasted deceleration of the global economy in the next years, and one of the projected lowest growth rates in recent decades.^5^ More recently, a new term has been used to define the current era, called the polycrisis^6^: multiple global crises that interact such that the overall effect exceeds the sum of each part. From a public health point of view, the worsening of socioeconomic conditions and deterioration of the main social determinants of health will substantially increase the morbidity and mortality in the affected populations, especially among the most at-risk individuals in LMICs.

Brazil, an LMIC as defined by the Organisation for Economic Co-operation and Development and the World Bank, led one of the most vigorous welfare state expansions among LMICs during the last 2 decades, implementing a public universal health care system and nationwide social protection programs that strongly reduced poverty and inequalities.^1^ The country developed one of the world’s largest conditional cash transfer (CCT) programs targeted at the poor population (Programa Bolsa Família [PBF]),^7,8^ one of the most comprehensive social pension programs for the population that is older and disabled (Beneficio de Prestacão Continuada [BPC]),^9^ and one of the most effective and robustly evaluated primary health care interventions designed to extend universal health coverage (Family Health Strategy [ESF]); a detailed description of these programs and their associations with health outcomes is provided in eAppendix 1 in Supplement 1.^10,11^ Moreover, Brazil is one of the LMICs where the COVID-19 pandemic had the strongest and most prolonged effect on the economy, with a rapid and sustained increase in poverty rates^12^ that hit its highest peak in more than a decade; and food insecurity that reached 60% of the population and left more than 15% in hunger (ie, severe food insecurity).^13^

The objective of this study was to evaluate the association of the Brazilian conditional cash transfers, social pensions, and primary health care with reductions of morbidity and mortality over almost 2 decades. This study also forecasted the mitigation potentials of these programs during the current global polycrisis and beyond.

## Methods

This cohort study used municipal-level data that does not directly involve human participants. Therefore, there was no requirement for informed consent or the approval of an institutional review board or ethics committee, in accordance with the Brazilian Data Protection Law and the Open Data Plan and data policy of the Brazilian Institute of Geography and Statistics. We followed the Strengthening the Reporting of Observational Studies in Epidemiology (STROBE) reporting guideline.

### Study Design

This study has a longitudinal ecological design, whereby municipalities (units of analysis) were observed annually from 2004 to 2019. This longitudinal dataset combines aggregated health, socioeconomic, and welfare programs’ data from several sources (eAppendix 1 in Supplement 1) from 2004 to 2019 for all Brazilian municipalities. Consistent with previous studies,^7,11^ 2548 municipalities with adequate vital statistics according to validated criteria^7,14^ were included in the analyses. The starting year was set to 2004 when PBF started, whereas both ESF and BPC were already implemented.^8^ Age-standardized all-cause mortality and hospitalization rates calculated annually for the entire population and by age group (<5 years of age, 5-29 years, 30-69 years, and ≥70 years) were used as dependent variables.

The annual coverage of PBF was calculated as the number of families enrolled in the PBF in a municipality divided by the number of eligible families (according to PBF criteria) in the same municipality (ie, target coverage).^7^ As in previous studies,^7,11,15^ we categorized the PBF coverage, and the coverage of all the interventions, to estimate the dose-response association related to increasing degrees of implementation of the interventions. Using previously established reference thresholds,^7,15^ we created 4 levels of PBF coverage: low (0%-29.9%), intermediate (30.0%-69.9%), high (70.0%-99.9%), and consolidated (≥100%). We calculated the annual ESF and the BPC coverage as the total number of individuals registered in each program divided by the municipality’s population. The ESF coverage was categorized, following previous studies,^11^ as null (0%), low (0.1%-29.9%), intermediate (30.0%-69.9%), and consolidated (70.0%-100%); whereas the BPC coverage, for which no previous references were available, was categorized using terciles: low (0%-32.9%), intermediate (33.0%-65.9%), and consolidated (66.0%-100%).

All relevant time-variant demographic-adjusted, socioeconomic-adjusted, and health care–adjusted variables according to the literature^2,7,11,15^ were included in the models: poverty rate, illiteracy rate, urbanization rate, fertility rate, percentage of households with inadequate garbage collection, and the number of physicians per 1000 population. A wide range of other covariates was also tested in a sensitivity analysis (eAppendix 2 in Supplement 1). As in previous studies, we dichotomized these covariables according to their median value over the period.^7,11,15^ Moreover, we included time dummy variables (for 2008-2009, 2013-2014, and 2015-2016) to adjust for major economic shocks that occurred in Brazil in the last 2 decades,^2,17^ and we tested several additional models with different time variable controls (eAppendix 2 in Supplement 1). Other unobserved time-invariant characteristics of the municipalities were adjusted by the fixed-effects term.^16^

### Statistical Analysis

#### Retrospective Analysis

We used negative binomial multivariable regression models with fixed-effects specification, which are consolidated methods for analysis with aggregate-level panel data and mortality rates and have been used in several evaluation studies of PBF and ESF.^2,11,15,17^ The negative binomial distribution was chosen because municipal mortality rates are characterized by overdispersion.^11,15,16,17^ The fixed-effects specification was selected based on the Hausmann test results, and because fixed-effects models allow to control for time-invariant unobserved variables associated with mortality and hospitalization rates, such as geographical, historical, or sociocultural aspects of each municipality.^16^ We reported rate ratios (RRs) and 95% CIs. All comparisons were 2-sided, and *P* < .05 was considered statistically significant. To evaluate the robustness of the estimates, we did several sensitivity analyses (eAppendix 2 in Supplement 1). Moreover, we performed triangulation analyses using difference-in-difference, with and without propensity-score matching.^16,18^ We used Stata version 14.0 (StataCorp) for database processing and analysis.

#### Forecast Analysis and Future Scenarios

To forecast the outcomes of the economic crisis and the mitigation potential of PBF, ESF, and BPC coverage changes, we used validated municipal-level microsimulation models. Microsimulation is among the most accurate forecasting methods because it allows the modeling of municipality-specific characteristics and their associated outcome probabilities.^19^ The modeling approach was derived from previous studies and was undertaken in 2 stages^17,20^: (1) we created a synthetic cohort of all Brazilian municipalities for the years 2020 to 2030, extrapolating and modeling each municipal-level independent variable from the 2004 to 2019 dataset; (2) we estimated the projection of the mortality rates using the independent variables as inputs in the same multivariate regression models used in the retrospective analysis, including the estimates of their associations.

In the first stage, 3 economic crisis scenarios (shorter, medium, and longer) were simulated through changes in poverty rates, using real data from nationwide socioeconomic microdata during the COVID-19 pandemic, and extrapolating poverty trends with the same methodology of previous studies^17,20^ (eAppendix 3 in Supplement 1). The economic crisis scenarios considered in this analysis were simulated as follows: (1) shorter economic crisis scenario: a milder and shorter economic crisis, with a moderate increase in poverty rate only for the years 2020 to 2022, and a reduction in the following years up to 2030; (2) medium economic crisis scenario: a more sustained increase in the poverty rates from 2020 to 2024, and a subsequent reduction; (3) longer economic crisis scenario: a sustained and longer increase of poverty rate over 7 years (from 2020 to 2026), followed by a gradual poverty reduction (eAppendix 3 in Supplement 1). Regarding the policy response to the economic crisis, 3 changes in PBF, ESF, and BPC coverage were simulated: mitigation scenario, baseline, and severe fiscal austerity scenario. The crisis-mitigation scenario was based on simulated increases of PBF, ESF, and BPC proportionally to the rise of poverty rates during the economic recession, and successive coverage reduction after the end of the crisis.^17,20^ The baseline scenario was derived from a validated model (already used in previous studies^17,20^) that projected the outcomes of the current fiscal austerity measures on the coverage of the 3 interventions (eAppendix 3 in Supplement 1). The severe austerity scenario was based on the reduction of PBF, ESF, and BPC proportional to the reduction of government expenditure on social protection (excluding cash transfer programs) observed from 2014 to 2019.^21^ For each outcome and each scenario, 10 000 Monte Carlo simulations were performed, allowing parameter values to vary in each simulation cycle according to their assumed underlying distribution. Further details of the modeling process in accordance with the international model reporting guidelines (ISPOR-SMSM)^22^ are provided in eAppendix 2 in Supplement 1. For the forecasting analyses, we used R version 4.1.2 (R Project for Statistical Computing). Statistical analyses were performed from September 2022 to February 2023.

## Results

### Retrospective Analysis

Among the 2548 Brazilian municipalities studied from 2004 to 2019, the mean (SD) age-standardized mortality rate decreased by 16.64% (from 6.73 [1.14] to 5.61 [0.94] deaths per 1000 population) (Table 1); the biggest decrease in mean (SD) mortality rate was 22.04% among children under 5 years of age (from 20.96 [11.21] to 16.34 [9.96] deaths per 1000 live births), and the lowest decrease was 3.53% among those aged 5 to 29 years (from 0.85 [0.49] to 0.82 [0.57] deaths per 1000 population). In the same period, the mean (SD) age-standardized hospitalization rates decreased by 13.45% (from 79.60 [24.94] to 68.89 [24.00] hospitalizations per 1000 population). From 2004 to 2019, the mean (SD) coverage of the PBF program increased by 103.35% (from 48.07% [23.73%] to 97.75% [9.42%]); the coverage of the ESF program increased by 40.46% (from 59.20% [38.03%] to 83.15% [25.03%]); and the coverage of the BPC program increased by 91.59% (from 1.07% [0.74%] to 2.05% [1.13%]). Overall, socioeconomic, health care, and living conditions improved during the study period.

**Table 1.** Mortality Rates, Hospitalization Rates, and Variables for Selected Municipalities (N = 2548)^a^

Variables	Mean (SD)				2004-2019, % change
	2004	2009	2014	2019	
Mortality rates					
Overall	6.73 (1.14)	6.14 (1.07)	5.82 (0.96)	5.61 (0.94)	−16.64
<5 y	20.96 (11.21)	18.5 (11.48)	16.15 (9.88)	16.34 (9.96)	−22.04
5-29 y	0.85 (0.49)	0.89 (0.52)	0.88 (0.57)	0.82 (0.57)	−3.53
30-69 y	6.48 (1.55)	5.91 (1.41)	5.56 (1.28)	5.33 (1.23)	−17.75
≥70 y	61.34 (13.72)	55.85 (12.6)	53.02 (10.38)	51.56 (10.07)	−15.94
Hospitalization rates					
Overall	79.6 (24.94)	72.06 (23.2)	68.57 (21.69)	68.89 (24)	−13.45
<5 y	447.11 (213.63)	406.69 (204.46)	347.37 (183.24)	366.85 (184.72)	−17.95
5-29 y	56.3 (20.65)	52.58 (17.66)	50.25 (16.83)	52.69 (19.59)	−6.41
30-69 y	80.96 (29.95)	73.41 (26.21)	69.89 (24.33)	70.47 (26.25)	−12.96
≥70 y	257.09 (101.26)	222.33 (92.2)	207.74 (82.03)	186.95 (76.83)	−27.28
Programs coverage					
PBF coverage, %	48.07 (23.73)	99.57 (3.75)	99.93 (1)	97.75 (9.42)	103.35
ESF coverage, %	59.2 (38.03)	71.7 (33.52)	78 (29.18)	83.15 (25.03)	40.46
BPC coverage, %	1.07 (0.74)	1.52 (0.96)	1.87 (1.06)	2.05 (1.13)	91.59
Other variables					
Poverty rate, %	19.51 (14.32)	11.38 (11.35)	5.97 (9.22)	6.01 (9.68)	−69.20
Illiteracy rate, %	12.18 (7.13)	10.37 (5.93)	8.96 (5.3)	6.75 (4.83)	−44.58
Urbanization rate, %	70.8 (19.63)	72.83 (18.91)	75.94 (18.42)	77.69 (18.3)	9.73
Fertility rate, %	3.42 (0.52)	3.19 (0.45)	2.93 (0.39)	2.77 (0.39)	−19.01
Households with proper garbage collection, %	72.66 (19.43)	80.64 (16.55)	86.52 (14.82)	89.84 (13.49)	23.64
Rate of physicians per 1000 population	0.75 (0.53)	0.77 (0.65)	0.86 (0.76)	1 (0.87)	33.33

Abbreviations: BPC, Benefício de Prestação Continuada; ESF, Family Health Program; PBF, Bolsa Familia Program.

^a^ The mortality and hospitalization rates are calculated per 1000 population of the municipality, except for the Under-5 category which is calculated by 1000 live births.

Table 2 shows the crude and adjusted associations of the overall mortality and hospitalization rates with the coverage levels of the 3 welfare programs (ie, PBF, ESF, and BPC). Consolidated coverages of PBF, ESF, and BPC were associated with the statistically significant reduction of age-standardized mortality rate (PBF: rate ratio [RR], 0.95 [95% CI, 0.94-0.96]; ESF: RR, 0.93 [95% CI, 0.92-0.94]; BPC: RR, 0.91 [95% CI, 0.91-0.92]). A dose-response association was present for PBF and BPC. Based on these models, we estimated how many overall deaths have been avoided over almost 2 decades (2004-2019) because of the implementation of the 3 programs comparing the real scenario with an alternative scenario where all independent variables had real trends and values, except for the coverage of all programs that was kept null during the period. According to this scenario’s comparison, the number of avoided overall deaths associated with PBF, ESF, and BPC implementation from 2004 to 2019 was 1 462 626 (95% CI, 1 332 128-1 596 924) (eAppendix 2 in Supplement 1).

**Table 2.** Association Between Mortality and Hospitalization Rates and PBF, ESF, and BPC Coverage^a^

Variables	RR (95% CI)			
	Mortality		Hospitalization	
	Crude	Adjusted	Crude	Adjusted
PBF coverage, %				
Low (0-29.9)	1 [Reference]	1 [Reference]	1 [Reference]	1 [Reference]
Intermediate (30-69.9)	0.98 (0.97-0.99)	0.98 (0.97-0.99)	0.98 (0.96-0.99)	0.97 (0.96-0.99)
High (70-99.9)	0.97 (0.96-0.98)	0.97 (0.96-0.98)	0.94 (0.93-0.95)	0.94 (0.93-0.96)
Consolidated (100)	0.94 (0.93-0.95)	0.95 (0.94-0.96)	0.90 (0.89-0.91)	0.91 (0.90-0.92)
ESF coverage, %				
Low (0)	1 [Reference]	1 [Reference]	1 [Reference]	1 [Reference]
Intermediate (0-29.9)	0.99 (0.99-1.00)	1.00 (0.99-1.01)	0.95 (0.94-0.96)	0.96 (0.95-0.97)
High (30.0-69.9)	0.93 (0.92-0.94)	0.93 (0.93-0.94)	0.96 (0.95-0.97)	0.96 (0.95-0.97)
Consolidated (70.0-100)	0.93 (0.92-0.93)	0.93 (0.92-0.94)	0.98 (0.97-0.99)	0.99 (0.98-1.00)
BPC coverage (terciles)				
Low (0-32.9)	1 [Reference]	1 [Reference]	1 [Reference]	1 [Reference]
Intermediate (33.0-65.9)	0.93 (0.93-0.94)	0.93 (0.93-0.94)	0.96 (0.96-0.97)	0.96 (0.96-0.97)
Consolidated (66.0-100)	0.90 (0.90-0.91)	0.91 (0.91-0.92)	0.86 (0.86-0.87)	0.88 (0.88-0.89)
Control variables				
Poverty rate, %	NA	1.02 (1.02-1.02)	NA	1.04 (1.03-1.04)
Illiteracy rate, %	NA	1.02 (1.01-1.02)	NA	1.03 (1.02-1.04)
Urbanization rate, %	NA	0.98 (0.98-0.99)	NA	0.95 (0.94-0.96)
Fertility rate, %	NA	1.01 (1.01-1.02)	NA	1.01 (1.00-1.01)
Households with proper garbage collection, %	NA	1.00 (0.99-1.01)	NA	0.97 (0.97-0.98)
Rate of physicians per 1000 population, %	NA	1.00 (1.00-1.01)	NA	0.99 (0.99-1.00)
Time shock binaries^b^	Yes	Yes	Yes	Yes

Abbreviations: BPC, Benefício de Prestação Continuada; ESF, Family Health Program; NA, not applicable; PBF, Bolsa Familia Program; RR, rate ratio.

^a^ RRs are from the fixed-effect negative binomial models for the association between mortality and hospitalization rates and PBF, ESF, and BPC coverage.

^b^ Time shocks are controls for specific years of economic crisis: 2008, 2013, and 2015. The total number of observations is 40 762: 2 548 municipalities and 16 years (from 2004 to 2019). Other time controls, including continuous-time and binary variables for other years, are in eAppendix in Supplement 1.

The age-stratified models (Table 3) showed a reduction of mortality associated with increasing coverage of the 3 interventions in almost all age groups. The largest observed reductions at the consolidated coverage level were in the mortality of the group aged younger than 5 years with an RR of 0.87 (95% CI, 0.85-0.89) for PBF, 0.90 (95% CI,0.87-0.93) for ESF, and 0.84 (95% CI, 0.82-0.86) for BPC. BPC also was found to have a larger negative association with mortality in the age groups of 30 to 69 years and 70 years and above. No statistically significant associations were encountered in mortality rates from transport accidents, used as control (eAppendix 2 in Supplement 1). All sensitivity analyses supported the robustness of the findings, and all triangulation analyses showed a high degree of confidence in the associations. They are presented in detail in eAppendix 2, eAppendix 3, and eAppendix 4 in Supplement 1.

**Table 3.** Association Between Mortality Rates and PBF, ESF, and BPC Coverage by Age Group^a^

Variables	Age group, RR (95% CI)			
	<5 y	5-29 y	30-69 y	≥70 y
PBF coverage, %				
Low (0-29.9)	1 [Reference]	1 [Reference]	1 [Reference]	1 [Reference]
Intermediate (30-69.9)	1.01 (0.98-1.04)	0.99 (0.96-1.02)	0.97 (0.96-0.98)	0.98 (0.96-0.99)
High (70-99.9)	0.96 (0.93-0.99)	0.99 (0.97-1.03)	0.97 (0.56-0.98)	0.97 (0.96-0.98)
Consolidated (100)	0.87 (0.85-0.89)	1.02 (0.99-1.04)	0.94 (0.93-0.95)	0.96 (0.95-0.97)
ESF coverage, %				
Low (0)	1 [Reference]	1 [Reference]	1 [Reference]	1 [Reference]
Intermediate (0-29.9)	1.01 (0.98-1.04)	0.99 (0.96-1.02)	1.01 (0.99-1.02)	0.99 (0.99-1.01)
High (30.0-69.9)	0.95 (0.92-0.97)	0.94 (0.92-0.97)	0.92 (0.92-0.93)	0.94 (0.93-0.94)
Consolidated (70.0-100)	0.90 (0.87-0.93)	0.94 (0.91-0.97)	0.93 (0.92-0.94)	0.93 (0.92-0.94)
BPC coverage, terciles				
Low (0-32.9)	1 [Reference]	1 [Reference]	1 [Reference]	1 [Reference]
Intermediate (33.0-65.9)	0.93 (0.92-0.94)	0.90 (0.87-0.91)	0.93 (0.92-0.93)	0.94 (0.94-0.94)
Consolidated (66.0-100)	0.84 (0.82-0.86)	0.96 (0.94-0.97)	0.89 (0.89-0.90)	0.92 (0.92-0.93)
Control variables				
Poverty rate, %	1.05 (1.03-1.06)	0.96 (0.95-0.97)	1.03 (1.02-1.03)	1.02 (1.01-1.02)
Illiteracy rate, %	1.05 (1.03-1.07)	1.05 (1.04-1.07)	1.02 (1.01-1.03)	1.02 (1.01-1.02)
Urbanization rate, %	0.93 (0.91-0.96)	0.94 (0.92-0.96)	0.99 (0.98-1.00)	0.99 (0.98-1.00)
Fertility rate, %	1.07 (1.05-1.09)	0.98 (0.97-0.99)	1.02 (1.02-1.03)	1.01 (1.00-1.01)
Households with proper garbage collection, %	0.95 (0.94-0.97)	1.01 (0.99-1.03)	1.00 (0.99-1.00)	0.99 (0.99-1.00)
Rate of physicians per 1000 population, %	0.98 (0.96-0.99)	1.00 (0.99-1.02)	1.00 (0.99-1.01)	1.01 (1.00-1.01)
Time shock binaries^b^	Yes	Yes	Yes	Yes

Abbreviations: BPC, Benefício de Prestação Continuada; ESF, Family Health Program; PBF, Bolsa Familia Program; RR, rate ratio.

^a^ RRs were from the fixed-effect negative binomial models by age group for the association between mortality rates and PBF, ESF, and BPC coverage.

^b^ Time shocks are controls for specific years of economic crisis: 2008, 2013, and 2015. The total number of observations is 40 762: 2 548 municipalities and 16 years (from 2004 to 2019). Other time controls, including continuous-time and binary variables for other years, are in eAppendix in Supplement 1.

### Forecast Analysis

The Figure shows 1 of the projections of increasing poverty rates, the shorter economic crisis scenario (eAppendix 3 in Supplement 1), up to 2030 alongside 3 scenarios of PBF, ESF, and BPC coverage: mitigation, baseline, and severe fiscal austerity. The Figure also shows the forecast of overall mortality rates for the respective austerity scenarios. In the mitigation scenario, mortality rates were projected to decrease over the next decade; in the baseline austerity, mortality rates were projected to slightly increase; and, in the severe austerity scenario, mortality rates were projected to significantly increase until the end of the period.

**Figure.**
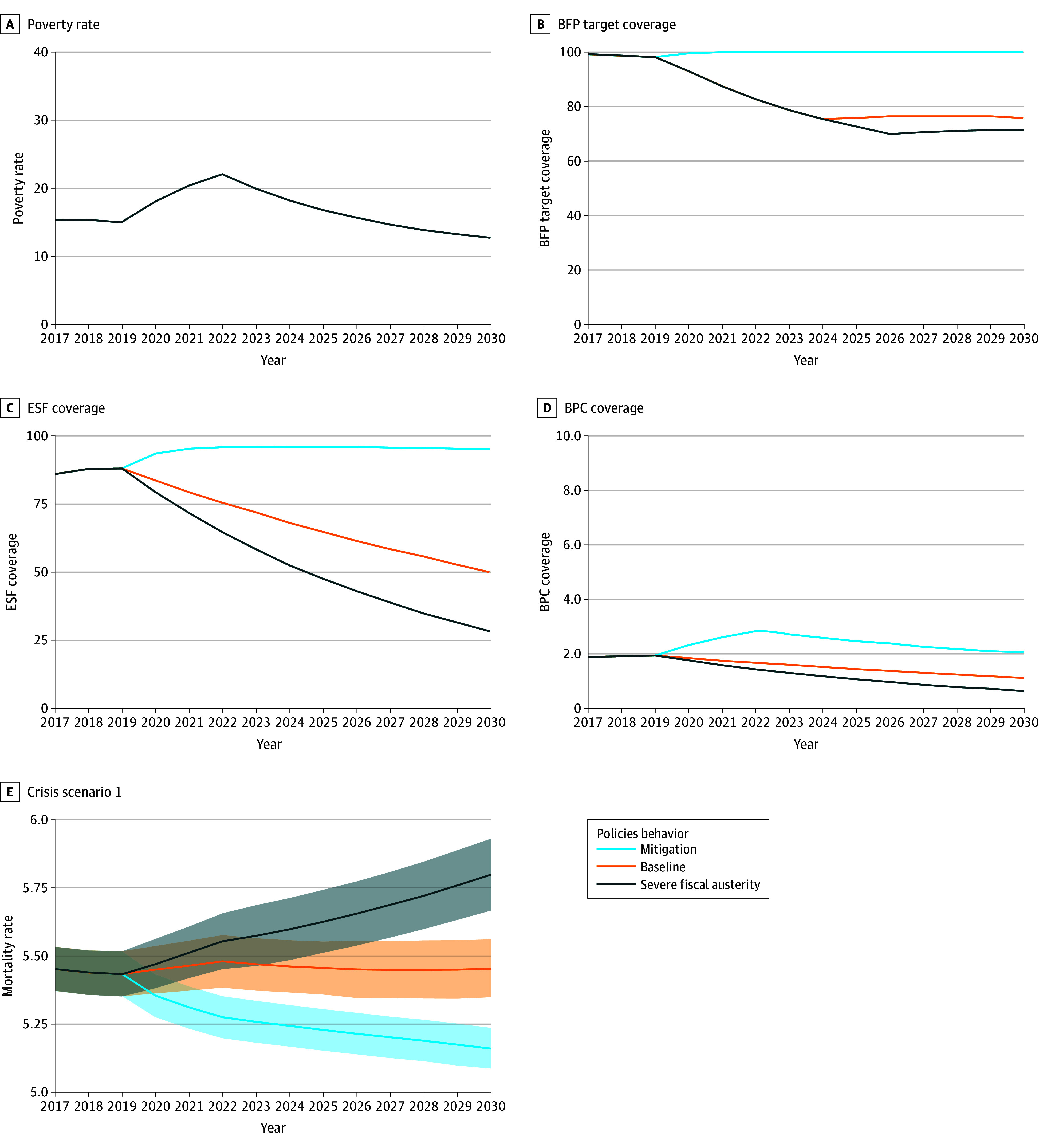
Scenarios of Poverty, Social Welfare Policies, and Overall Mortality Projections, 2020-2030 The mortality rates are per 1000 people. BFP indicates Bolsa Familia Program; BPC, Benefício de Prestação Continuada; ESF, Family Health Strategy.

In Table 4, RRs are reported for the comparison between scenarios. In 2030, for overall mortality, the RR between mitigation and baseline was projected to be 0.95 (95% CI, 0.93-0.96), whereas the RR between mitigation and severe austerity was projected to be 0.89 (95% CI, 0.87-0.91). These projections corresponded to an estimated 660 661 (95% CI, 571 301-750 621) averted deaths from 2020 to 2030 if mitigation strategies were implemented for the 3 interventions instead of keeping their coverages at the baseline levels. When compared with severe fiscal austerity scenarios, mitigation strategies were projected to avert 1 305 359 (95% CI, 1 163 659-1 449 256) deaths during the same period. For mortality among children younger than 5 years, increasing coverages to mitigate the results of the crisis was projected to correspond to an RR of 0.75 (95% CI, 0.73-0.78) for an estimated 105 544 (95% CI, 99 510-111 671) averted deaths among children younger than 5 in comparison with the baseline scenario; or to a projected RR of 0.65 (95% CI, 0.62-0.66) and an estimated 182 531 (95% CI, 172 600-192 523) averted deaths among children younger than 5 years when compared with the severe fiscal austerity scenario. Regarding hospitalization rates, mitigation compared with the baseline scenario was projected to correspond to an RR of 0.98 (95% CI, 0.96-0.99) and an estimated 4 697 468 (95% CI, 4 006 945-5 412 836) averted hospitalizations. Numbers are higher when the mitigation scenario is compared with the severe austerity scenarios, with a projected RR of 0.96 (95% CI, 0.94-0.98) and an estimated 6 593 224 (95% CI, 5 534 591-7 651 327) averted hospitalizations. The RRs and the number of averted deaths associated with other economic crisis scenarios and combinations of policy responses are comparable in magnitude and are reported in eAppendix 3 in Supplement 1.

**Table 4.** Projected Avoidable Deaths From Comparison of Forecasted Mitigation, Baseline, and Austerity Scenarios, 2020 to 2030

Year	Projected mortality, rate ratio (95% CI)	
	Mitigation vs baseline	Mitigation vs austerity
**Overall mortality**		
2020	0.98 (0.98-0.99)	0.98 (0.97-0.99)
2025	0.96 (0.95-0.97)	0.93 (0.91-0.96)
2030	0.95 (0.93-0.96)	0.89 (0.87-0.91)
Projected avoidable deaths, No. (95% CI)	660 661 (571 301-750 621)	1 305 359 (1 163 659-1 449 256)
**Child mortality**		
2020	0.92 (0.91-0.93)	0.91 (0.90-0.99)
2025	0.83 (0.81-0.84)	0.70 (0.68-0.74)
2030	0.75 (0.73-0.78)	0.65 (0.62-0.66)
Projected avoidable deaths, No. (95% CI)	105 544 (99 510-111 671)	182 531 (172 600-192 523)

## Discussion

Our study found that cash transfers, social pensions, and primary health care were associated with a decrease in hospitalization and mortality rates over the last 2 decades in Brazil. We also found that the implementation of these programs may have prevented more than 1.4 million overall deaths between 2004 and 2019. Moreover, using robust forecasting methods, we show that increased coverage of these programs (as a mitigation strategy for the current economic crisis) could avert up to 1.3 million deaths by 2030, including 182 531 deaths among children under age 5, when compared with a response based on fiscal austerity measures. To the best of our knowledge, this is the first study that performs a nationwide combined evaluation of cash transfers, social pensions, and primary health care for such a long period in an LMIC and uses these estimates to forecast their mitigation potentials during the global polycrisis.

The PBF is a nationwide conditional cash transfer program in Brazil that aims to reduce poverty and its consequences through cash transfers to economically at-risk households, conditioned to health, nutritional, and educational requirements. The ESF is a primary health care program within the Unified Health System that provides community-based care to families and households in selected communities through multidisciplinary teams composed of physicians, nurses, and community health workers. The ESF teams reinforce the promotion, prevention, protection, diagnosis, treatment, rehabilitation, harm reduction, palliative care, and health surveillance of the Unified Health System in Brazil. The BPC is a noncontributory social pension program implemented to ensure subsistence and independence, and also guarantee the reduction of poverty and risk of the older (aged above 65 years) and disabled population.

A detailed description of the mechanisms that could explain the large consequence of each intervention on health outcomes are shown in eAppendix 1 in Supplement 1. Regarding conditional cash transfers, although previous studies have also shown that they are associated with reduced child, maternal, and disease-specific mortalities,^7,15,23,24^ to our knowledge, none has ever evaluated its association with overall, adult, and older adults’ mortality. There are several mechanisms through which these outcomes may occur. First, the income transferred to poor and extremely poor families improves families’ nutrition and living conditions (ie, the income effect).^24^ Second, they condition beneficiaries to a minimum usage of health services for child and maternal health (ie, the conditionality effect). Additionally, conditional cash transfers are also able to improve a wide range of socioeconomic factors that affect health, such as improved education, reduced inequalities, and social exclusion.^24^ Regarding PBF in Brazil, its association with reduced overall morbidity and mortality is expected due to its proven effect on child and maternal mortality,^7,15^ in particular for diarrheal diseases and malnutrition, and its potential to reduce incidence and mortality from all poverty-related causes.^8,23^

Unlike conditional cash transfers, which only attend to a target population, primary health care programs are designed to attend to the entire population. The channels by which primary health care is associated with reduced mortality and hospitalization are by increasing preventive care, early detection, and first-level treatment of illnesses.^25^ In Brazil, the large expansion of ESF was 1 of the factors responsible for the reduction of hospitalizations for ambulatory-sensitive conditions,^10,26^ and for the decreases in child and adult mortality from several causes, including heart and cerebrovascular diseases.^11^

LMICs, especially in Latin America and the Caribbean, are facing a demographic transition toward older populations, and the implementation of social or noncontributory pensions has been crucial to maintaining and improving the well-being of older individuals.^27^ Our findings on the positive association of social pensions with reduction of mortality and hospitalization are supported by previous studies showing that they increase access to health care and medications,^28^ better economic conditions and reduced poverty,^29^ and improved nutrition.^30^ The reduction of mortality and hospitalization rates associated with the BPC across all age groups can be explained by the positive externality of the benefits transferred to older individuals or individuals with disabilities in other household members. Studies have found evidence of improved health outcomes for children living in the same households with social pension beneficiaries.^31^

Despite having lower coverage, the stronger results found for BPC compared with the PBF and ESF may be associated with its level of generosity in terms of the value of benefits (fixed at a minimum salary) compared with the PBF, which transfers only a small fraction of the minimum salary. The stronger association of the PBF and BPC with reductions in hospitalizations compared with mortality is plausible, given that their health influences are indirectly manifested through the conditionality of health care usage. The stronger association of the ESF with mortality reductions than with hospitalizations is also plausible given that the program could, on one side, reduce avoidable hospitalizations, but, on the other side, also increase the referral to second and higher levels of health care in populations not previously attended by any health care service.^26^ Economic crises, irrespective of the cause, are characterized by higher poverty and unemployment, loss of purchasing power due to inflation, and increased inequality, the effects of which are exacerbated in LMICs. The most common political response to these economic downturns in LMICs is the implementation of fiscal austerity measures to reduce public debt, and this is often translated into the reduction of social protection and health care services. In Brazil, the capacity of the Brazilian government to mitigate the adverse consequences of economic crises is currently constrained by the long-term fiscal austerity measure of the Constitutional Amendment 95 (EC95), which restricts the growth of federal expenditure on social protection and health care, bringing to a progressive reduction of their coverage.^2,17,20^ Our forecast analysis suggests that austerity measures that reduce the coverage of social welfare programs during economic crises are detrimental to the health of the population. In turn, these negative health outcomes could impair the economy through the permanent loss of human capital (avoidable deaths), and the increased expenditure on health care (avoidable hospitalizations).^32^ These economic implications of such health outcomes deserve further empirical investigations.

### Limitations

This study has limitations. First, the selection of municipalities with a higher quality of vital information in the retrospective analyses, which strengthened the internal validity of the study, could limit its generalizability. However, this has been a common strategy of previous nationwide studies using the same data and analytic design,^7,11,15^ also because the majority of the Brazilian population and all regions of the country are represented in the municipalities under study. To verify our external validity, we fitted our models with data from all Brazilian municipalities and found similar and significant results, indicating that our main findings could be generalized for the entire country. Another limitation is the uncertainty of the forecasted scenarios of economic crisis: because the economic and political situation is still volatile, poverty rates could continue to increase even after macroeconomic indicators are improving, similar to what happened in the previous Brazilian economic crisis.^2,12,17^ For that reason, different economic crisis scenarios were forecasted, showing consistent comparison estimates between alternative policy responses.

## Conclusion

Our study found that conditional cash transfers, social pensions, and primary health care were associated with reduced morbidity and mortality in Brazil. Moreover, all of our forecasted scenarios suggest that a prompt expansion of these programs could represent a rapid and effective mitigation response to the adverse health consequence of the current global polycrisis, in comparison with fiscal austerity measures that could cause large numbers of preventable deaths.

## Notes

Supplement 1. **eAppendix 1.** Summary of Social Programs, Data Sources and General Methodology **eAppendix 2.** Retrospective Analysis **eAppendix 3.** Forecasting Analysis **eAppendix 4.** Triangulation Analyses
eReferences

Supplement 2.
Data Sharing Statement
