# Supplementary material for: Current and Projected Mortality and Hospitalization Rates Associated With Conditional Cash Transfer, Social Pension, and Primary Health Care Programs in Brazil, 2000-2030
Source: JAMA Netw Open. 2024 Apr 22;7(4):e247519. doi: 10.1001/jamanetworkopen.2024.7519 (PMC11036142; doi:10.1001/jamanetworkopen.2024.7519)
Supplement: Supplement 1. — eAppendix 1. Summary of Social Programs, Data Sources and General Methodology eAppendix 2. Retrospective Analysis eAppendix 3. Forecasting Analysis eAppendix 4. Triangulation Analyses eReferences [file jamanetwopen-e247519-s001.pdf]

Supplemental Online Content

Aransiola TJ, Cavalcanti D, Ordoñez JA, et al. Current and projected mortality and hospitalization rates associated with conditional cash transfer, social pension, and primary health care programs in Brazil, 2000-2030. *JAMA Netw Open.* 2024;7(4):e247519. doi:10.1001/jamanetworkopen.2024.7519

eAppendix 1. Summary of Social Programs, Data Sources and General Methodology

eAppendix 2. Retrospective Analysis

eAppendix 3. Forecasting Analysis

eAppendix 4. Triangulation Analyses

eReferences

This supplemental material has been provided by the authors to give readers additional information about their work.

© 2024 Aransiola TJ et al. *JAMA Network Open.*

eAppendix 1. SUMMARY OF SOCIAL PROGRAMS, DATA SOURCES AND GENERAL METHODOLOGY

1. Background - social welfare state programs in Brazil

1.1. Bolsa Família Program (BFP)

The Bolsa Família is the Brazilian Conditional Cash Transference (CCT) program that aims to attenuate the effects of poverty through a minimum cash transfer for beneficiary families, and to break the intergenerational cycle of poverty through investment in education and health conditionalities.[1]This important socio-economic intervention was established in 2004 by Law № 10,836, of January 9, 2004, with the last monetary restatement modified by Decree № 9,396, of May 30, 2018, in which households are eligible for the program if their per capita income is equal to or less than R$89.00 (approximately US$19 at current 2022 prices) or if they are poor families with income up to R$ 178.00 (approximately US$ 38 at current 2022 prices) and one member is a child up to 17 years old or a pregnant woman (or a woman who just gave birth).[2]

In the health area, conditionalities concern the monitoring of vaccination and nutritional surveillance of children, as well as prenatal care of pregnant women and the puerperium, and should be attended at the FHS units. Some studies have shown the effect of BFP on child morbidity and mortality[1], including its synergistic effects with FHS[3], and on other health outcomes associated with poverty, such as tuberculosis, leprosy, mortality from suicides, and homicides.[4]

The BFP is one of the largest CCT in the world with more than 13.9 million families benefiting throughout Brazil, and was recently remodeled and called Axílio Brasil Program (ABP) by Law № 14,284, of December 29, 2021. Basically, this new program maintains the conditionalities and general structure of the BFP, but it increases the coverage and the value transferred through changes in the criteria of poverty and extreme poverty; where families earning up to R$105.00 per capita (approximately US$22 at current 2022 prices) and up to R$210.00 (almost US$45 at current 2022 prices) are considered extremely poor and poor, respectively. No studies were found that directly evaluate the ABP, however studies that simulated scenarios of increased coverage of BFP show improvements in health outcomes.[5]

1.2. Family Health Strategy (FHS)

The "Estratégia Saúde da Família" (FHS; Family Health Strategy – previously called Family Health Program), is the primary vehicle for achieving Universal Health Coverage (UHC) within the SUS and is one of the largest Primary Health Care (PHC) programmes of the world. Implemented since 1994, the FHS encompasses key principles of PHC including community-based care, multi-disciplinary teams, and a focus on health prevention and promotion, composed of physicians, nurses, and community health workers to provide basic health care and preventive services to families and households in selected communities. The FHS teams reinforces the promotion, prevention, protection, diagnosis, treatment, rehabilitation, harm reduction, palliative care and health surveillance of the Unified Health System (SUS) in Brazil.

The FHS coverage has expanded from 6.6% in 1998 to 63.7% in 2016 (covering 123 million people)[6], and encompasses key principles of PHC including community-based care, multi-

© 2024 Aransiola TJ et al. *JAMA Network Open.*

disciplinary teams, and a focus on health prevention and promotion.[7] Prior studies associated expanding FHS coverage with reductions in infant mortality,[8] adult mortality from conditions amenable to healthcare[7], cardiovascular mortality[9] and health inequalities.[10] Despite the large amount of evidence of its effectiveness, according to recent forecasting studies performed by the proponents of the project, austerity measures implemented in the country could affect future FHS coverage, especially in the poorest areas, and be responsible for a large number of avoidable child and adult deaths in the next years.[6]

1.3. Benefício de Prestação Continuada (BPC)

The Conditional Welfare Benefit (BPC) is a non-contributory social protection program that was implemented by the 1988 Brazilian Federal Constitution to ensure the welfare of the elderly (above age 65) and disabled population. The program guarantees a minimum wage payment to the target population whose family income per capita does is below a quarter of the minimum salary. The objective is to ensure the subsistence and independence, and also guarantee the reduction of poverty and vulnerability of the elderly and disabled population. By May 2020, about 2.08 and 2.57 million of the elderly and disabled population are assisted by the BPC program[1]. The government bodies responsible for the management and operationalization of the BPC program are the Ministry of Citizenship (previously called "Ministry of Social Development and Fight Against Hunger" – MDS), National Social Assistance Secretariat (SNAS), Ministry of Social Security (MPS), National Social Security Institute (INSS), and the Social Assistance Reference centers (CRAS).

The BPC program encompasses two important social welfare initiatives designed for the disabled beneficiaries: the BPC School Program and the BPC Employment Program. The BPC School Program aims to enable access and follow-up the school attendance record of the disabled beneficiaries below the age of 18. The BPC Employment Program helps the beneficiaries between age 16 and 64 who desire to work but encounter barriers to gain access into the labor force through vocational trainings and educational courses. The channel thorough with this initiative is executes is the Program for Access to Technical Education and Employment (PRONATEC) managed by the Ministry of Education.

The participation in the BPC program is frequently (every two years) reassessed to ensure that the beneficiaries continue to meet the eligibility condition regarding family income, and also occasionally regarding the deficiency status, i.e., deficient beneficiaries who recovered from the reported illness and have sufficient income are suspended are eliminated from the program. Moreover, the benefits from the BPC are exclusive to the beneficiary and not transferrable.

1.4. Mechanisms of the effect of BFP, FHS, and BPC on health outcomes

There are several mechanisms through which the BFP program affect health outcomes. First, the BFP conditions beneficiaries to a minimum usage of health services for child and maternal health, i.e., the conditionality effect.[11,12] Second, the income transferred to poor and extremely poor families improves the nutrition and living conditions of these families, i.e, the income effect[13]. Third, long-term exposure to the health conditionality of the BFP promote behavior changes and adherence of beneficiary families towards health care[14]. The main channel through which the FHP reduces mortality and hospitalization are through the increase of preventive care, early detection, and treatment of severe illnesses[1,5]. Moreover, FHP strengthens the health impact of the BFP through the follow-up of health conditionalities of the BFP by health workers[1,15]. Previous studies show that the BPC affect health outcomes through increased access to healthcare and

medications[14,16;] reduced labor and exposure to hazardous working condition[17,18]; better economic conditions, i.e., reduced poverty[19], and; improved nutrition[18]. Regarding the strong mitigating effect of the BPC on under-5 mortality, studies have found evidence of improved health outcomes of children living in the same households with social pension beneficiaries.[20–22] It is also important to recall that the BPC attends to both the elderly population and deficient population irrespective of age.

eFigure 1: Mechanisms linking the Social Pension Program, Bolsa Familia Program and the Family Health Program to health outcomes.

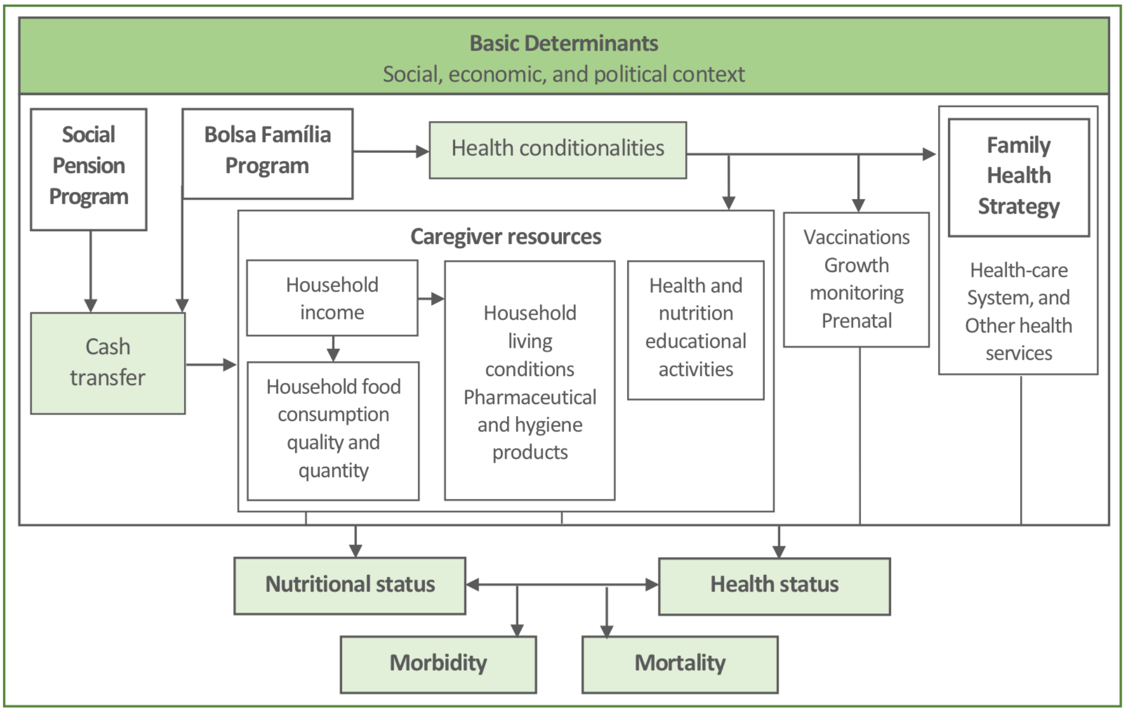

© 2024 Aransiola TJ et al. *JAMA Network Open.*

2. Dataset

2.1. Data sources

The data used in this study were obtained from various governmental platforms detailed in eTable 1. All the variables used in this study are aggregated to the municipal level. However, the data for some variables were not available for specific years and municipalities, therefore we performed linear interpolation as detailed in Section 2.2. of this supplementary document.

eTable 1. Data Sources and description

Variable	Years	Units of analysis	Source	Link
Mortality and Morbidity	2000 to 2019	Municipality	DATASUS - SIM	https://datasus.saude.gov.br/mortalidade-desde-1996-pela-cid-10
Population estimates	2000 to 2019	Municipality	IBGE – Census	https://www.ibge.gov.br/
Municipal population age sex-race distributions	2000 to 2019	Municipality	IBGE – Census	https://www.ibge.gov.br/
Livebirth	2000 to 2019	Municipality	DATASUS - SINASC	https://datasus.saude.gov.br/nascidos-vivos-desde-1994
Gini index	2000 and 2010, 2001-2019	State and Municipality	IBGE - Census; IBGE - PNAD; and IBGE - PNADC	https://sidra.ibge.gov.br/pesquisa/censo-demografico/demografico-2010/inicial
Illiteracy rate (Percentual of people older than 15 years who are illiterate)	2000 and 2010, 2001-2019	State and Municipality	IBGE - Census; IBGE - PNAD; and IBGE - PNADC	https://sidra.ibge.gov.br/pesquisa/pnadct/tabelas
Poverty rate (Poor population estimate)	2000 and 2010, 2001-2019	State and Municipality	IBGE - Census; IBGE - PNAD; and IBGE - PNADC	https://www.ibge.gov.br/estatisticas/sociais/populacao/9127-pesquisa-nacional-por-amostra-de-domicilios.html?=&t=downloads
Percentual of household with piped water	2000 and 2010, 2001-2019	State and Municipality	IBGE - Census; IBGE - PNAD; and IBGE - PNADC	https://sidra.ibge.gov.br/pesquisa/censo-demografico/demografico-2010/inicial

© 2024 Aransiola TJ et al. *JAMA Network Open.*

Percentual of household with sewage/sanitation	2000 and 2010, 2001-2019	State and Municipality	IBGE - Census; IBGE - PNAD; and IBGE - PNADC	https://sidra.ibge.gov.br/pesquisa/pnadct/tabelas
Bolsa Família program coverage	2004 to 2019	Municipality	MDS	https://aplicacoes.mds.gov.br/sagi/vis/data3/data-explorer.php
Family Health Strategy coverage	2000 to 2019	Municipality	DATASUS - DAB	https://datasus.saude.gov.br/cnes-equipes-de-saude
Benefício de Prestação Continuada Program coverage (Brazilian non-contributory pension benefit)	2004 to 2019	Municipality	MDS	https://aplicacoes.mds.gov.br/sagi/vis/data3/data-explorer.php
Hospital bed rate (beds per 1,000 people)	2000 to 2019	Municipality	DATASUS - CNES	https://datasus.saude.gov.br/cnes-recursos-fisicos
Doctor rate (Physicians per 1,000 people)	2000 to 2019	Municipality	DATASUS - CNES	https://datasus.saude.gov.br/cnes-equipes-de-saude

Note: DATASUS - Department of Informatics of the Unified Health System (Departamento de Informática do Sistema Único de Saúde); SIM - Mortality Information System (Sistema de Informações sobre Mortalidade); DAB - Department of Primary Care (Departamento de Atenção Básica); CNES - National Register of Health Establishments (Cadastro Nacional de Estabelecimentos de Saúde; SINACS - Live Birth Information System (Sistema de Informações sobre Nascidos Vivos); IBGE - The Brazilian Institute of Geography and Statistics (Instituto Brasileiro de Geografia e Estatística); MDS - The Ministry of Social Development and Fight against Hunger (Ministério do Desenvolvimento Social e Combate à Fome); PNAD - (Pesquisa Nacional por Amostra de Domicílios); PNADC - Continuous PNAD Quarterly (Pesquisa Nacional por Amostra de Domicílios Contínua).

2.2. *Interpolation and extrapolation method*

Longitudinal data collection regarding socioeconomic variables is not frequent at the municipal level given the high cost of conducting surveys in Brazil. Therefore, in most cases empirical studies resort to the linear interpolation of data; settle with the use of census cross-sectional data, or; use state-level data. In Brazil, the most robust municipal-level socio-economic data are obtained from the national census, which was last conducted in the year 2000 and 2010. However, the Brazilian National Household Sample Survey (PNAD), in which the smallest unit of aggregation is the state, is conducted every year.

In this study, instead of performing conventional linear interpolation of the two data points (2000 and 2010 for each municipality, we perform a more robust form of linear interpolation whereby,

in addition to the linear interpolated data, we input variation extracted from state-level data. This procedure can be simply understood as the linear interpolation of municipal data adjusted by state-level information.

This procedure is carried out in four steps:

a) Given the complete state-level information, the first step is then to estimate an Ordinary Least Squares (OLS) linear regression of each variable of interest as a function of time $t = 2000, \ldots, 2018$. Then, we obtain the residuals e_t, as follows:

$$\hat{e}_{jt} = y_{jt} - z_{jt} \text{, where } z_t = \widehat{\beta_{0j}} + \widehat{\beta_{1j}}t \quad (1),$$

with $t = 2000, \ldots, 2019$ and $\widehat{\beta_{0\iota t}}, \widehat{\beta_{1\iota t}}$ being the regression coefficients estimated by the OLS method. Therefore, 20 residuals will be obtained for each state, corresponding to the 2000-2019 period.

b) For the municipality level, using the census information available (years 2001 and 2010), the data was inter-extrapolated following the next sub-steps,

i. We calculate the linear equation between the two data points $(2000, y_{ij2000})$ and $(2010, y_{ij2010})$, where y_{ijt} denotes the value of y at the i-th municipality from the j-th state at the time t. This equation will be of the form,

$$z_{ijt} = \beta_{0ij} + \beta_{1ij}t \quad (2)$$

ii. Subsequently, we calculate the z_{ijt} values for $t = 2001, \ldots, 2009, \ldots, 2011, \ldots, 2018$ using equation (2) (the values corresponding to t = 2000 and t= 2010 are already available from the census, so they do not need to be imputed).

iii. Finally, we impute y_{ijt} for $t \neq 2000, 2010$ as:

$$y_{ijt} = z_{ijt} + e_{it} \quad (3)$$

where e_{it} corresponds to the state residual calculated from the regression in (1).

2.2.1. Discussion and Results

The proposed method imputes the state behavior observed in PNAD microdata to annual municipal fluctuations in non-census periods, that is, 2001 to 2009, and 2011 to 2019. With this it is possible to capture important effects that occurred in this period, such as breaks in trends and temporal shocks caused by the Brazilian economic crises of 2008, 2013 and 2015[5]; and its consequences on several socioeconomic variables, such as the increase in income inequality and poverty of families in Brazil[23].

A total of 892,134 values were generated for 9 socioeconomic variables, in 5,507 municipalities over 18 years with this method. The interpolated variables were Illiteracy rate, urbanization rate, household infrastructure (garbage, sewage, piped water), and inequality and income variables (family income, Gini index, poverty rate and extreme poverty rate). Some of these variables were used as control variables for the models, none of the outcome variables (mortality and morbidities) or exposure variables (BPC, BFP and FHS) were interpolated. In the end, it was observed that the interpolated variables improved the control and precision of the retrospective and predictive models.

eAppendix 2. RETROSPECTIVE ANALYSIS

3. Empirical methods

3.1. Negative binomial regression – Fixed Effects

We estimate Fixed Effect models using the negative binomial method to retrospectively evaluate and forecast the impact of each welfare social policy on health outcomes. The equation which describes the linear relationship between the health outcomes (mortality and hospitalization rates) and covariates is given by:

$$Log\,Y_{it} = \alpha_i + \sum_{q=1}^{3} \beta_q BFP_{qit} + \sum_{r=4}^{6} \beta_r ESF_{rit} + \sum_{s=6}^{7} \beta_s BPC_{sit} + \beta_8 T_{2008} + \beta_9 T_{2013} + \beta_{10} T_{2015} + \sum_{k=11}^{16} \beta_k X_{kit},$$

where:

t refers to the year, i refers to an individual municipality, and q, r, s are indexes representing the categories of each intervention

Y_{it} are the different welfare state variables (mortality and hospitalization rates for age group) observed at the municipality i in year t,

BFP_{qit} are the dummies representing the BFP coverage categories (4 groups) observed at the municipality i in year t with a coefficient of β_q,

ESF_{rit} are the dummies representing the FHS coverage categories (4 groups) observed at the municipality i in year t with a coefficient of β_r,

BPC_{sit} are the dummies representing the BPC coverage categories (terciles) observed at the municipality i in year t with a coefficient of β_s,

$T_{2008}, T_{2013}, T_{2015},$ are dummy variables representing previous crisis events with coefficients $\beta_8, \beta_9, \beta_{10}$ respectively,

X_{kit} represents different control covariates, each one with a coefficient of β_k (Poverty, illiteracy, fertility and doctors rates and percentage of households with garbage collection).

α_i is the fixed effect (time-invariant) term for each municipality.

© 2024 Aransiola TJ et al. *JAMA Network Open.*

4. Results

4.1. Descriptive Analysis

In this section (from eFigures 2 – 11), we present figures that describe the temporal dynamics of the BFP, FHS, and BPC, alongside the health outcomes (hospitalization and mortality rates by age group and overall) from the year 2004 to 2019. During this period, the overall rate of mortality and hospitalization rates reduced expressively, except for after the 2015 crisis, especially for the under-5 and above-70 age groups.

Regarding social welfare programs, eFigures 12-15, showed that the coverages of the BFP, FHS, and BPC increased steadily from 2004 to 2018, although at a diminishing rate due to the long-term fiscal policy measure adopted since the 2013 economic crisis. In the year 2019, the reduction of the coverages of these programs was clear. eFigure 15 shows the average rate of mortality and hospitalization rates over the BFP, FHS, and BPC coverage levels (in increasing order). In most cases, an inverse or negative correlation is perceptible between coverage levels and health outcomes.

eFigure 2: Overall mortality rate, 2004-2019.

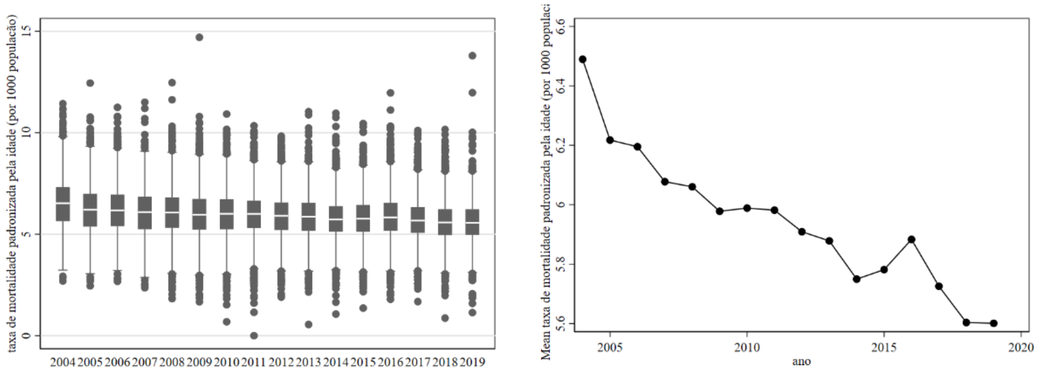

eFigure 3: Under-five mortality rate (U5MR), 2004-2019.

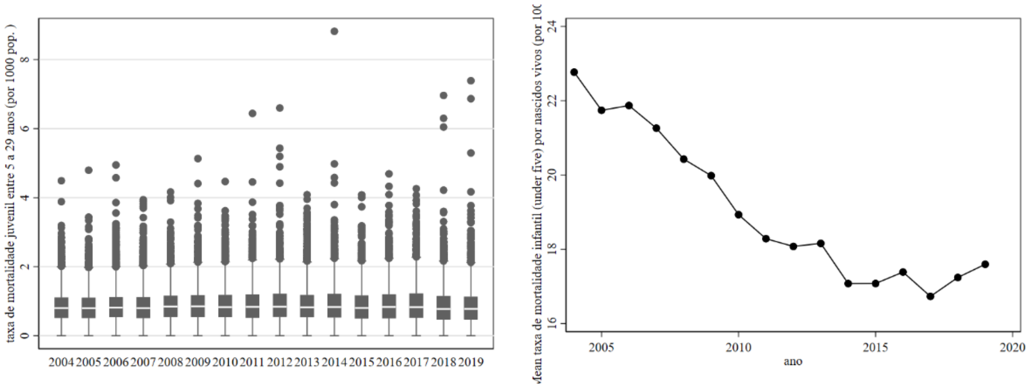

© 2024 Aransiola TJ et al. *JAMA Network Open.*

eFigure 4: Mortality rate among individuals from age 5 to 29, 2004-2019.

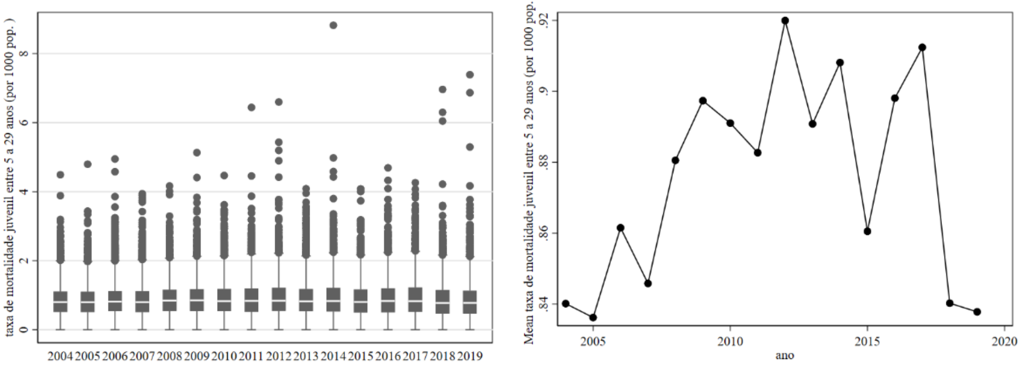

eFigure 5: Mortality rate among individuals from age 30 to 69, 2004-2019.

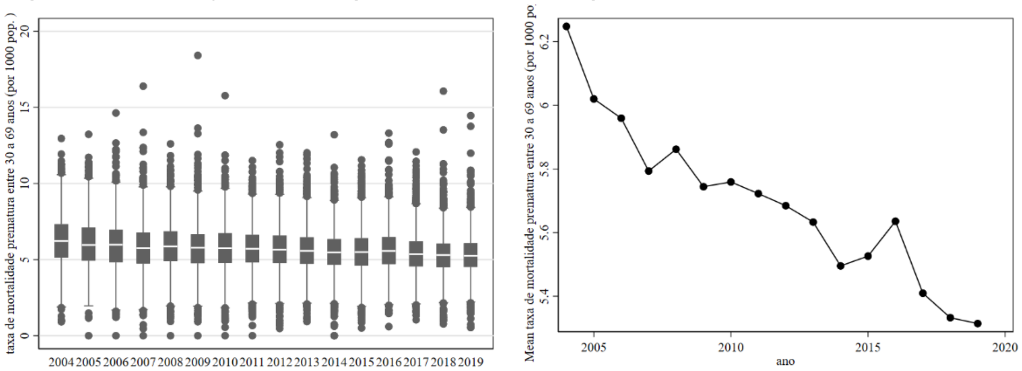

eFigure 6: Mortality rate among individuals above age 70, 2004-2019.

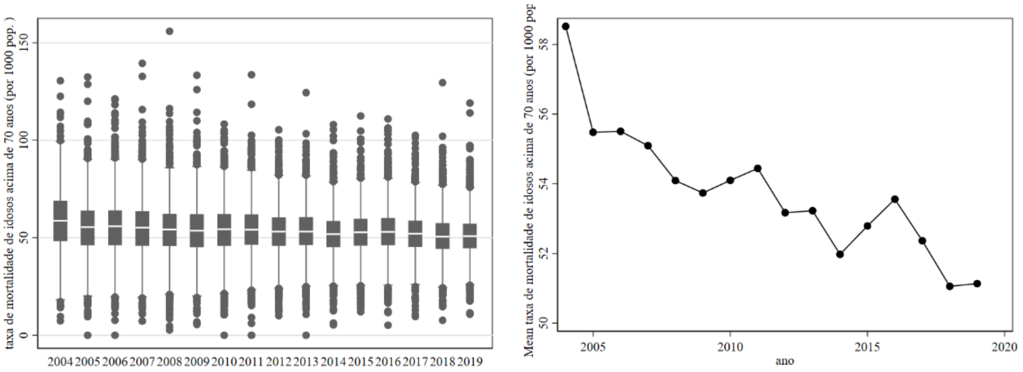

© 2024 Aransiola TJ et al. *JAMA Network Open.*

eFigure 7: Hospitalization rate, 2004-2019.

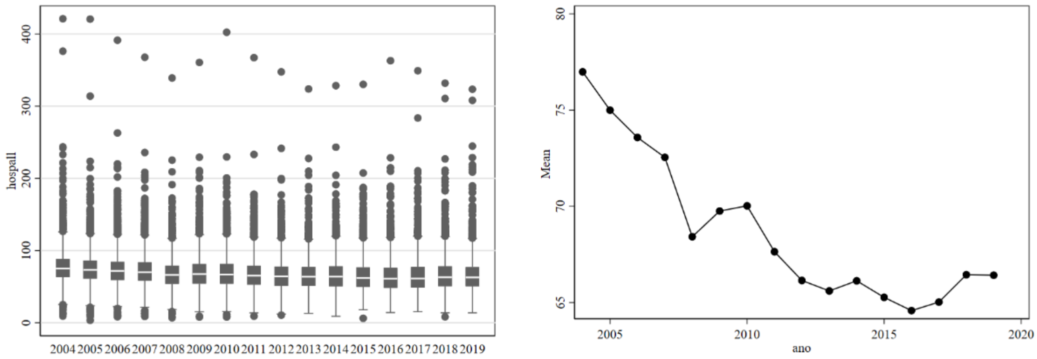

eFigure 8: Under-five hospitalization rate (U5HR), 2004-2019.

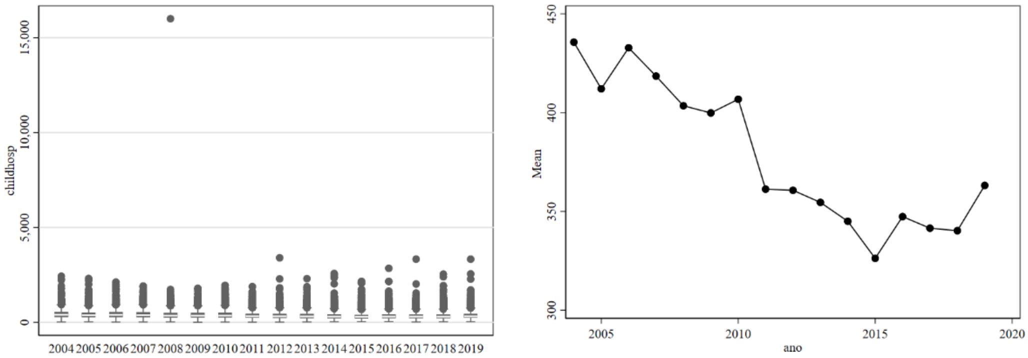

eFigure 9: Hospitalization rate among individuals from age 5 to 29, 2004-2019.

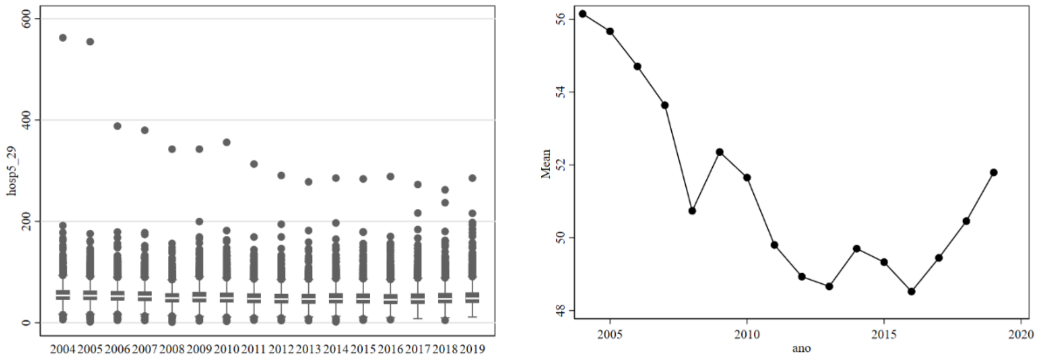

© 2024 Aransiola TJ et al. *JAMA Network Open.*

eFigure 10: Hospitalization rate among individuals from age 30 to 69, 2004-2019.

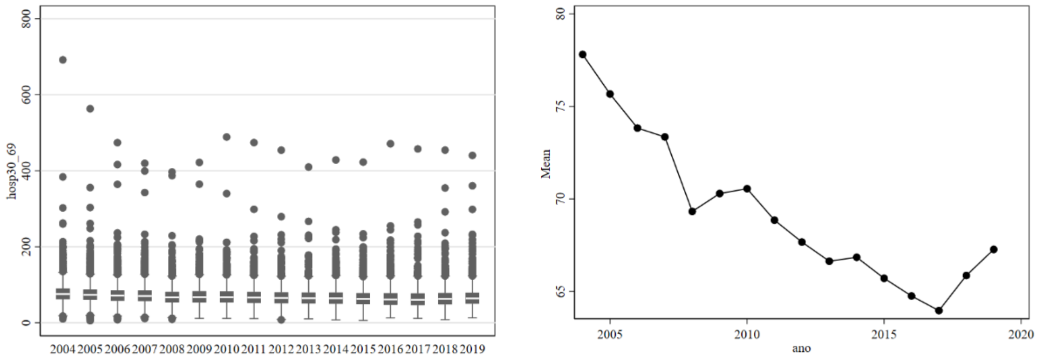

eFigure 11: Hospitalization rate among individuals above age 70, 2004-2019.

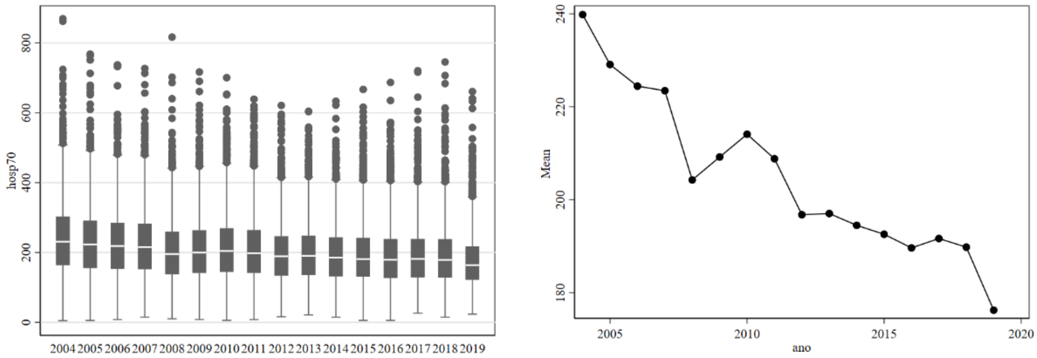

eFigure 12: Coverage of the Bolsa Família Program (target population), 2004-2019.

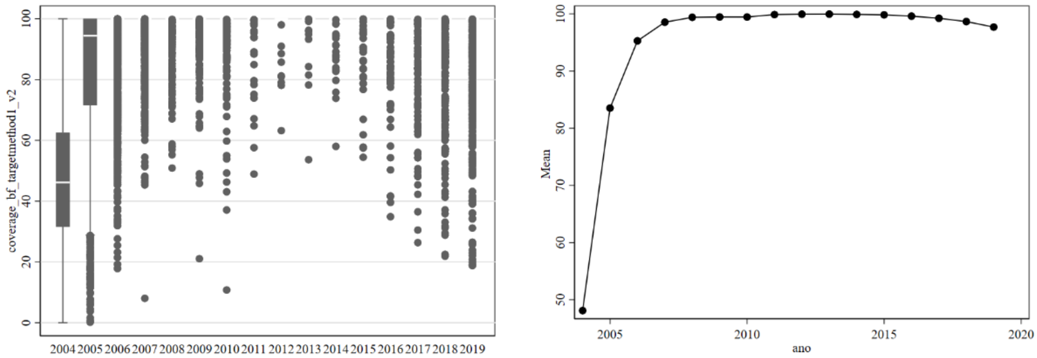

© 2024 Aransiola TJ et al. *JAMA Network Open.*

eFigure 13: Coverage of the Family Health Program (target population), 2004-2019.

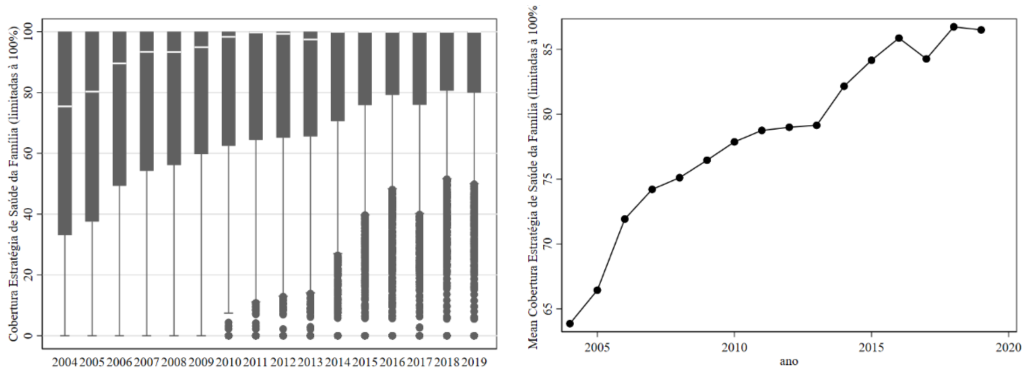

eFigure 14: Coverage of the Continuous Cash Benefits Program (population), 2004-2019.

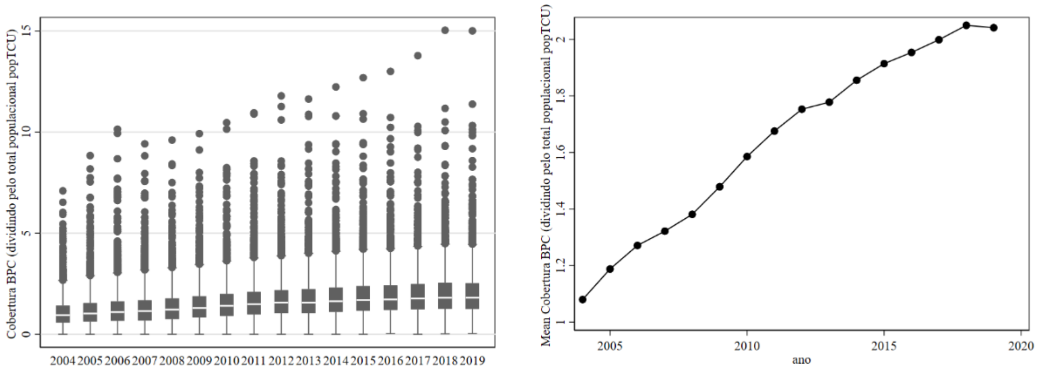

© 2024 Aransiola TJ et al. *JAMA Network Open.*

eFigure 15: Average mortality and hospitalization rate by the level of coverage of the BFP, FHS, and CCB social welfare programs, 2004-2019.

Mortality Hospitalization

Bolsa Família Program

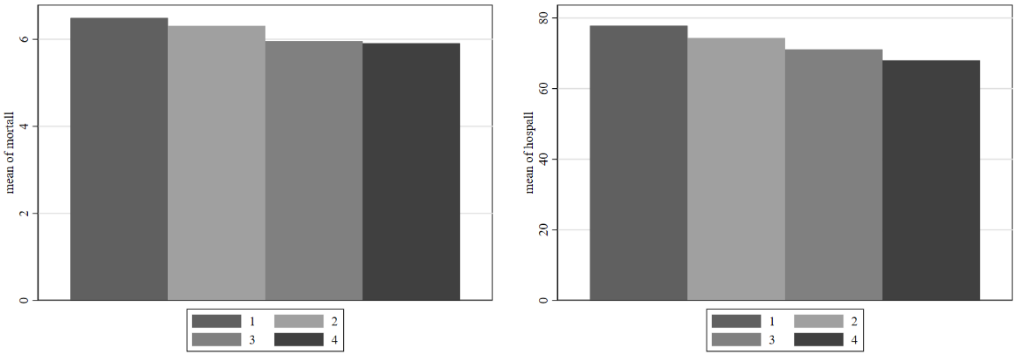

Family Health Program

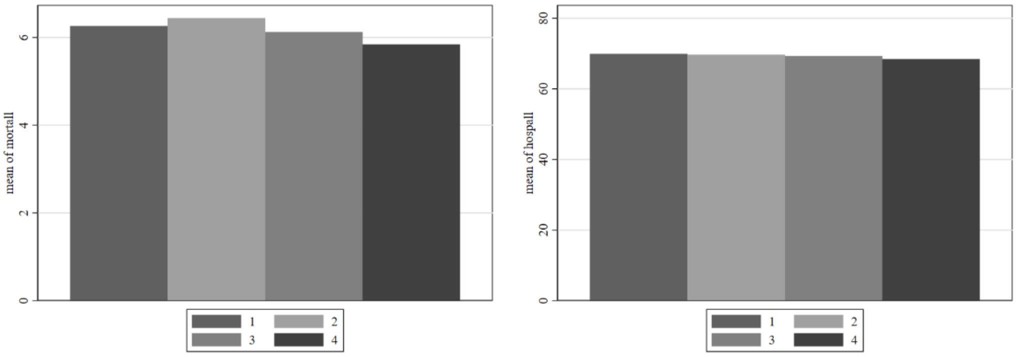

Continous Cash Benefit Program

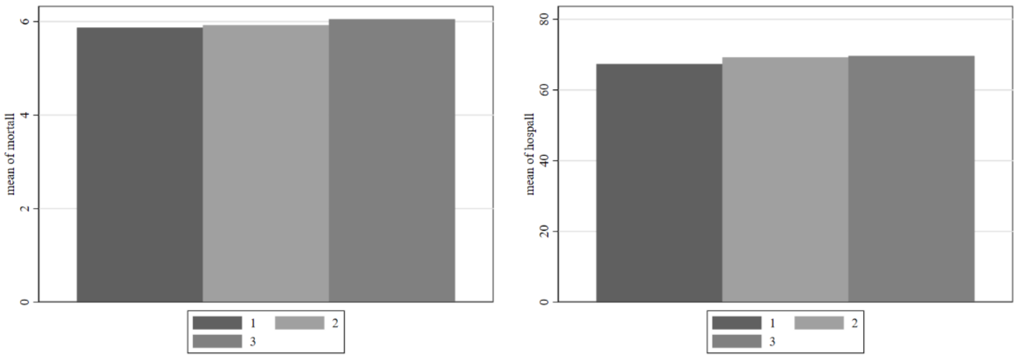

eTable 2: Descriptive Statistics of the dependent and independent variables, 2004-2019.

**Variables**	**Mean**	**Variance**	**Standard deviation**
Mortality rate	5,94	overall	1,15
		between	0,73
		within	0,89
Hospitalization rate	68,72	overall	24,11
		between	19,92
		within	13,59
Poverty	14,69	overall	15,73
		between	14,34
		within	6,47
GINI	47,35	overall	7,85
		between	6,78
		within	3,96
Illitracy	11,87	overall	8,12
		between	7,71
		within	2,56
Urbanization	70,38	overall	20,15
		between	19,80
		within	3,74
Bolsa Família Program (BFP)	23,86	overall	17,16
		between	16,05
		within	6,08
Family Health Program (FHS)	94,40	overall	46,86
		between	40,09
		within	24,26
Continuous Cash Benefit (BPC)	1,65	overall	1,12
		between	1,01
		within	0,47
Physicians	0,74	overall	0,64
		between	0,56
		within	0,32
Nurse rate	0,61	overall	0,37
		between	0,25
		within	0,28
Hospital bet rate	2,29	overall	2,37
		between	2,23
		within	0,80

© 2024 Aransiola TJ et al. *JAMA Network Open.*

4.2. Sensitivity Analyses

We performed several fitting and sensitivity tests to ensure that the results presented in this study regarding the effect of social protection and healthcare policies on mortality and hospitalization are robust.

First, we performed the Hausman test for the overall mortality and hospitalization model specifications (Table 2 of the manuscript) to choose between the Fixed and Random Effect models, and the former was deemed more appropriate. Subsequently, we tested the goodness of fit using the Log-likelihood, AIC, and BIC, which all confirmed that the adjusted models for mortality and hospitalization rates yield better results compared to unadjusted models (see eTable 3).

Second, to verify the effect of the filter for adequate information on the results we estimated the same models without excluding municipalities without adequate data, i.e., using a total of 5,507 municipalities. The results presented in eTable 4 indicate that the significance and direction of effect are preserved for most variables. However, note that the gradient of the effect of the PBF and FHS is inverted as coverage levels increase, implying that the mitigating effect of these programs reduces as the coverage levels reduce. This may be attributed to data bias since most of the vulnerable municipalities are the ones with poor data quality.

Third, to test the time frame delimitation from 2004 to 2019 we use the data from 2000 to2019 (eTable 5 and 6) although acknowledging that the PBF was only implemented in 2004. Still, the results from both samples are aligned.

Fourth, to further test the stability of our model specification, we added more dependent variables to the models. Note that the new variables were excluded from the final models presented in the manuscript since similar controls have already been included in the model. eTable 7 shows that the cumulative addition of these new redundant variables did not affect the previous results, i.e., the estimates are stable.

Fifth, we used dichotomized variables following previous studies. However, in eTable 8, we show that the direction of effect found for PBF, FHS, and BPC are aligned when continuous variables are used.

Sixth, poverty is correlated to most socioeconomic variables. In our case, the PBF and BPC are not just correlated but also conditioned to poverty rates, which may compromise the result found for these programs. Therefore, in eTable 9 we estimate the overall models for mortality and hospitalization with and without the control for poverty rate and concluded that there is little or no effect of collinearity of poverty on our results.

Seventh, we tested four additional models with zero and different time shock controls (eTable 10) and compared the results to our final overall models. Nevertheless, the overall results continue stable.

Eight, we compared the overall results obtained using the Negative Binomial regression to that from Poisson regressions using the Log-likelihood, AIC, and BIC information criteria (see eTable 11). The test results showed that the Negative Binomial method yields better estimates. This is reasonable since the negative binomial regression takes the data overdispersion into account, compared to the Poisson which does not.

© 2024 Aransiola TJ et al. *JAMA Network Open.*

Ninth, we estimated the overall results including interaction terms between PBF, FHS, and BPC to verify if the synergies between them affected our results. The results, presented in eTable 12, indicates that the interaction between the programs does not affect the general conclusions drawn regarding the welfare programs.

Tenth, in eTable 13 we estimated the same models using as outcome age-standardized mortality due to transport accidents (ICD10 codes V01-V99), which is considered a negative control because BFP, FHS, and BPC are not expected to influence this group of causes and do not include actions for its prevention.

In other words, given that our results withstood the vast number of sensitivity tests performed, we conclude that the results and conclusions drawn in this study are robust and stable.

eTable 3: Empirical tests

	Mortality		Hospitalization	
	Without controls	With controls	Without controls	With controls
N. of observations<br>(n. of variables)	40762<br>(12)	40762<br>(18)	40762<br>(12)	40762<br>(18)
Log likelihood	-146185	-146030.7	-253104.3	-252796.8
Akaike information criterion (AIC)	292394.0	292097.4	506232.5	505629.6
Bayesian information criterion (BIC)	292497.4	292252.4	506335.9	505784.7
Hausman test	χ^2=1249.58; p-value= 0.000			

© 2024 Aransiola TJ et al. *JAMA Network Open.*

eTable 4 – Empirical results for mortality and hospitalization models without filter for adequate information, 2004-2019, Brazil. (Incidence-Rate Ratios -- IRR)

	Mortality		Hospitalization	
	Without controls	With controls	Without controls	With controls
PBF(0-30)	1	1	1	1
PBF(30-70)	1.004	1.003	1.003	1.019***
	[0.997,1.012]	[0.996,1.010]	[0.990,1.016]	[1.006,1.033]
PBF(70-100)	0.987***	0.983***	0.964***	0.992
	[0.980,0.994]	[0.976,0.990]	[0.951,0.976]	[0.979,1.005]
PBF(100)	0.990***	0.982***	0.889***	0.943***
	[0.984,0.997]	[0.976,0.989]	[0.879,0.900]	[0.932,0.955]
FHS(0)	1	1	1	1
FHS(0-30)	0.996	0.995*	0.882***	0.890***
	[0.991,1.002]	[0.990,1.001]	[0.874,0.891]	[0.881,0.899]
FHS(30-70)	0.936***	0.935***	0.966***	0.964***
	[0.931,0.941]	[0.930,0.940]	[0.956,0.976]	[0.954,0.974]
FHS(70-100)	0.958***	0.951***	1.084***	1.082***
	[0.952,0.964]	[0.946,0.957]	[1.072,1.095]	[1.071,1.094]
BPC(0-33)	1	1	1	1
BPC(33-66)	0.925***	0.925***	0.905***	0.915***
	[0.923,0.928]	[0.922,0.927]	[0.900,0.910]	[0.911,0.920]
BPC(66-100)	0.895***	0.890***	0.844***	0.876***
	[0.891,0.898]	[0.887,0.894]	[0.838,0.850]	[0.870,0.882]
Poverty		1.011***		1.040***
		[1.007,1.015]		[1.033,1.048]
Illiteracy		0.974***		1.075***
		[0.970,0.979]		[1.066,1.084]
Urbanization		1.022***		0.848***
		[1.015,1.029]		[0.839,0.857]
Fertility		0.983***		1.055***
		[0.979,0.987]		[1.047,1.062]
Garbage		1.026***		0.946***
		[1.021,1.030]		[0.938,0.954]
Physicians		1.008***		0.940***
		[1.005,1.011]		[0.935,0.946]
Year binaries	Yes	Yes	Yes	Yes

Note: Incidence-Rate Ratio (IRR) coefficients; Confidence intervals in parentheses; Time shocks are controls for specific years of economic crisis

© 2024 Aransiola TJ et al. *JAMA Network Open.*

-- 2008, 2013, and 2015; ***, ** and * denote significance at 1%, 5%, and 10% respectively. The total number of observations is 88,106 -- 5,507 municipalities and 16 years (from 2004 to 2019).

eTable 5 – Empirical results for mortality models with filter for adequate information, 2000-2019, Brazil. (Incidence-Rate Ratios -- IRR)

	Overall	Under 5	5 - 29	30 - 69	Above 70
PBF(0-30)	1	1	1	1	1
PBF(30-70)	0.981***	1.011	1002	0.975***	0.979***
	[0.972,0.991]	[0.979,1.045]	[0.971,1.033]	[0.963,0.988]	[0.968,0.992]
PBF(70-100)	0.977***	0.965**	0.994	0.971***	0.981***
	[0.968,0.986]	[0.935,0.997]	[0.964,1.025]	[0.960,0.983]	[0.970,0.993]
PBF(100)	0.946***	0.895***	0.988	0.937***	0.956***
	[0.938,0.954]	[0.869,0.923]	[0.960,1.016]	[0.926,0.947]	[0.946,0.967]
FHS(0)	1	1	1	1	1
FHS(0-30)	0.996	1008	0.986	1.002	0.998
	[0.989,1.004]	[0.978,1.038]	[0.960,1.013]	[0.992,1.012]	[0.989,1.007]
FHS(30-70)	0.933***	0.959***	0.925***	0.924***	0.941***
	[0.926,0.940]	[0.930,0.988]	[0.900,0.951]	[0.915,0.933]	[0.932,0.950]
FHS(70-100)	0.927***	0.922***	0.921***	0.928***	0.930***
	[0.919,0.935]	[0.892,0.954]	[0.894,0.949]	[0.918,0.938]	[0.921,0.940]
BPC(0-33)	1	1	1	1	1
BPC(33-66)	0.929***	0.927***	0.897***	0.924***	0.937***
	[0.926,0.933]	[0.914,0.939]	[0.887,0.909]	[0.919,0.928]	[0.933,0.941]
BPC(66-100)	0.897***	0.851***	0.935***	0.876***	0.906***
	[0.892,0.902]	[0.834,0.869]	[0.918,0.952]	[0.869,0.882]	[0.900,0.912]
Poverty	1.014***	1.042***	0.967***	1.020***	1.012***
	[1.009,1.018]	[1.026,1.059]	[0.954,0.980]	[1.014,1.026]	[1.006,1.017]
Illiteracy	1.013***	1.045***	1.052***	1.020***	1.011***
	[1.007,1.018]	[1.023,1.067]	[1.033,1.071]	[1.012,1.028]	[1.004,1.018]
Urbanization	0.999	0.946***	0.936***	1004	0.993
	[0.990,1.008]	[0.915,0.979]	[0.909,0.964]	[0.992,1.017]	[0.982,1.005]
Fertility	1.014***	1.044***	0.978**	1.024***	1.014***
	[1.008,1.019]	[1.024,1.064]	[0.961,0.995]	[1.016,1.031]	[1.007,1.021]
Garbage	1.003	0.991	1.063***	1.003	0.989***
	[0.997,1.009]	[0.969,1.013]	[1.042,1.085]	[0.994,1.011]	[0.981,0.996]
Physicians	0.998	0.995	0.996	0.997	0.995
	[0.993,1.003]	[0.976,1.015]	[0.979,1.014]	[0.991,1.004]	[0.989,1.002]
Year binaries	Yes	Yes	Yes	Yes	Yes

Note: Incidence-Rate Ratio (IRR) coefficients; Confidence intervals in parentheses; Time shocks are controls for specific years of economic crisis -- 2008, 2013, and 2015; ***, ** and * denote significance at 1%, 5%, and 10% respectively. The total number of observations is 40.762 -- 1.788 municipalities and 20 years (from 2000 to 2019).

eTable 6 – Empirical results for mortality models with filter for adequate information, 2000-2019, Brazil. (Incidence-Rate Ratios -- IRR)

Variables	Overall	Under 5	5 - 29	30 - 69	Above 70
PBF(0-30)	1	1	1	1	1
PBF(30-70)	0.966***	1.030**	0.977***	0.950***	0.929***
	[0.953,0.980]	[1.006,1.055]	[0.963,0.992]	[0.936,0.965]	[0.911,0.947]
PBF(70-100)	0.930***	1.029**	0.936***	0.909***	0.908***
	[0.918,0.943]	[1.006,1.054]	[0.923,0.949]	[0.895,0.922]	[0.891,0.925]
PBF(100)	0.906***	1.022**	0.910***	0.882***	0.873***
	[0.895,0.918]	[1.000,1.045]	[0.898,0.922]	[0.870,0.894]	[0.858,0.889]
FHS(0)	1	1	1	1	1
FHS(0-30)	0.961***	0.897***	0.964***	0.966***	0.992
	[0.950,0.972]	[0.880,0.914]	[0.952,0.976]	[0.954,0.978]	[0.977,1.007]
FHS(30-70)	0.963***	0.924***	0.978***	0.951***	0.937***
	[0.953,0.975]	[0.906,0.942]	[0.966,0.990]	[0.940,0.963]	[0.923,0.951]
FHS(70-100)	0.992	0.952***	1.008	1002	0.968***
	[0.980,1.005]	[0.933,0.973]	[0.994,1.021]	[0.989,1.015]	[0.952,0.984]
BPC(0-33)	1	1	1	1	1
BPC(33-66)	0.962***	0.993	0.980***	0.940***	0.910***
	[0.957,0.967]	[0.984,1.002]	[0.974,0.985]	[0.935,0.946]	[0.903,0.917]
BPC(66-100)	0.896***	0.901***	0.934***	0.865***	0.836***
	[0.888,0.903]	[0.889,0.914]	[0.926,0.942]	[0.857,0.873]	[0.827,0.846]
Poverty	1.041***	1.062***	1.039***	1.032***	1.028***
	[1.034,1.048]	[1.050,1.073]	[1.032,1.046]	[1.025,1.039]	[1.019,1.038]
Illiteracy	1.039***	1.085***	1.043***	1.046***	1.040***
	[1.031,1.048]	[1.070,1.099]	[1.034,1.052]	[1.036,1.055]	[1.028,1.052]
Urbanization	0.940***	0.824***	0.950***	0.932***	0.902***
	[0.928,0.952]	[0.808,0.840]	[0.937,0.962]	[0.919,0.944]	[0.887,0.917]
Fertility	1.014***	1.036***	1.015***	1.018***	1.028***
	[1.006,1.022]	[1.023,1.049]	[1.007,1.023]	[1.009,1.027]	[1.017,1.039]
Garbage	0.971***	0.953***	0.972***	0.975***	0.935***
	[0.963,0.980]	[0.939,0.966]	[0.963,0.981]	[0.965,0.984]	[0.923,0.946]
Physicians	0.990***	0.949***	0.992**	0.985***	0.967***
	[0.983,0.997]	[0.937,0.960]	[0.984,0.999]	[0.977,0.993]	[0.958,0.976]
Year binaries	Yes	Yes	Yes	Yes	Yes

© 2024 Aransiola TJ et al. *JAMA Network Open.*

Note: Incidence-Rate Ratio (IRR) coefficients; Confidence intervals in parentheses; Time shocks are controls for specific years of economic crisis -- 2008, 2013, and 2015; ***, ** and * denote significance at 1%, 5%, and 10% respectively. The total number of observations is 40.762 -- 1.788 municipalities and 20 years (from 2000 to 2019).

eTable 7 – Mortality models with additional variables.

	Model I	Model II	Model III	Model IV	Model V
	Overall	I + Education (degrees)	II + Living conditions	III + Inequality	IV + Nurse rate
PBF(0-30)	1	1	1	1	1
PBF(30-70)	1.003	1.002	1.002	1.002	1.001
	[0.996,1.010]	[0.995,1.010]	[0.995,1.009]	[0.995,1.009]	[0.994,1.009]
PBF(70-100)	0.983***	0.983***	0.982***	0.981***	0.981***
	[0.976,0.990]	[0.976,0.990]	[0.975,0.989]	[0.975,0.988]	[0.974,0.987]
PBF(100)	0.982***	0.980***	0.977***	0.974***	0.975***
	[0.976,0.989]	[0.973,0.986]	[0.971,0.984]	[0.968,0.981]	[0.968,0.982]
FHS(0)	1	1	1	1	1
FHS(0-30)	0.995*	0.995*	0.996*	0.995**	0.996
	[0.990,1.001]	[0.990,1.000]	[0.990,1.001]	[0.989,1.000]	[0.991,1.002]
FHS(30-70)	0.935***	0.935***	0.936***	0.935***	0.939***
	[0.930,0.940]	[0.930,0.940]	[0.931,0.941]	[0.930,0.940]	[0.934,0.944]
FHS(70-100)	0.951***	0.949***	0.949***	0.947***	0.954***
	[0.946,0.957]	[0.943,0.955]	[0.944,0.955]	[0.942,0.953]	[0.949,0.960]
BPC(0-33)	1	1	1	1	1
BPC(33-66)	0.925***	0.925***	0.924***	0.924***	0.929***
	[0.922,0.927]	[0.923,0.927]	[0.922,0.927]	[0.922,0.927]	[0.927,0.932]
BPC(66-100)	0.890***	0.891***	0.889***	0.888***	0.897***
	[0.887,0.894]	[0.887,0.894]	[0.886,0.892]	[0.885,0.891]	[0.894,0.901]
Poverty	1.011***	1.011***	1.014***	1.013***	1.009***
	[1.007,1.015]	[1.008,1.015]	[1.010,1.018]	[1.009,1.016]	[1.006,1.013]
Illiteracy	0.974***	0.977***	0.976***	0.978***	0.976***
	[0.970,0.979]	[0.972,0.982]	[0.972,0.981]	[0.973,0.983]	[0.971,0.980]
Urbanization	1.022***	1.020***	1.018***	1.015***	1.017***
	[1.015,1.029]	[1.013,1.027]	[1.011,1.025]	[1.008,1.022]	[1.010,1.024]
Fertility	0.983***	0.988***	0.988***	0.989***	0.984***
	[0.979,0.987]	[0.984,0.992]	[0.984,0.992]	[0.985,0.993]	[0.980,0.988]
Garbage	1.026***	1.021***	1.017***	1.017***	1.019***
	[1.021,1.030]	[1.016,1.026]	[1.012,1.022]	[1.012,1.021]	[1.014,1.023]
Physicians	1.008***	1.008***	1.007***	1.007***	1.011***
	[1.005,1.011]	[1.005,1.011]	[1.004,1.011]	[1.003,1.010]	[1.008,1.015]
Pri. Educ		1.027***	1.025***	1.023***	1.026***

© 2024 Aransiola TJ et al. *JAMA Network Open.*

		[1.023,1.030]	[1.021,1.029]	[1.020,1.027]	[1.023,1.030]
High school		0.998	0.996*	0.995***	0.996**
		[0.994,1.002]	[0.993,1.000]	[0.991,0.998]	[0.992,0.999]
Sewage			0.993***	0.993***	0.992***
			[0.990,0.995]	[0.991,0.995]	[0.990,0.995]
Water			0.981***	0.982***	0.979***
			[0.977,0.985]	[0.978,0.986]	[0.975,0.983]
GINI				0.941***	0.939***
				[0.935,0.947]	[0.933,0.945]
Nurse rate					0.974***
					[0.972,0.976]
Year binaries	Yes	Yes	Yes	Yes	Yes

Note: Pri. Educ and Sec. Educ is the percentage of population with primary and high school attainment; Sewage is the percentage of households with adequate sewage collection; Water is the percentage of households with access to treated water; GINI is the income inequality index, and; Nurse rate is the rate of nurses per 1000 population.

© 2024 Aransiola TJ et al. *JAMA Network Open.*

eTable 8 - Empirical results for mortality and hospitalization models using continuous variables (with filter for adequate information), 2004-2019, Brazil.

	Mortality		Hospitalization	
	Without controls	With controls	Without controls	With controls
BPF	1.000***	0.999***	0.998***	0.999***
	[1.000,1.000]	[0.999,0.999]	[0.998,0.998]	[0.999,0.999]
FHS	0.999***	0.999***	1.003***	1.003***
	[0.999,0.999]	[0.999,1.000]	[1.003,1.003]	[1.003,1.003]
BPC	0.958***	0.959***	0.876***	0.969***
	[0.956,0.960]	[0.957,0.962]	[0.873,0.879]	[0.965,0.973]
Poverty		0.994***		1.003***
		[0.993,0.995]		[1.002,1.004]
Illiteracy		0.995***		0.996***
		[0.994,0.995]		[0.996,0.996]
Urbanization		0.999***		0.990***
		[0.999,1.000]		[0.990,0.990]
Fertility		1.164***		1.252***
		[1.156,1.173]		[1.238,1.267]
Garbage		0.938***		0.867***
		[0.936,0.940]		[0.862,0.871]
Physicians		1.003***		1.003***
		[1.002,1.003]		[1.003,1.004]
Year binaries	Yes	Yes	Yes	Yes
Constant	0.00417***	0.00352***	0.000820***	0.000639***
	[0.00410,0.00424]	[0.00339,0.00365]	[0.000809,0.000832]	[0.000608,0.000673]

© 2024 Aransiola TJ et al. *JAMA Network Open.*

eTable 9 – Empirical results for mortality and hospitalization models with and without controls for poverty rate, 2004-2019, Brazil.

	Mortality		Hospitalization	
	With poverty	Without poverty	With poverty	Without poverty
PBF(0-30)	1	1	1	1
PBF(30-70)	1.003	1.003	1.019***	1.019***
	[0.996,1.010]	[0.996,1.010]	[1.006,1.033]	[1.006,1.033]
PBF(70-100)	0.983***	0.983***	0.992	0.990
	[0.976,0.990]	[0.976,0.990]	[0.979,1.005]	[0.977,1.003]
PBF(100)	0.982***	0.980***	0.943***	0.938***
	[0.976,0.989]	[0.974,0.987]	[0.932,0.955]	[0.927,0.950]
FHS(0)	1	1	1	1
FHS(0-30)	0.995*	0.996	0.890***	0.891***
	[0.990,1.001]	[0.990,1.001]	[0.881,0.899]	[0.881,0.900]
FHS(30-70)	0.935***	0.935***	0.964***	0.966***
	[0.930,0.940]	[0.930,0.940]	[0.954,0.974]	[0.956,0.976]
FHS(70-100)	0.951***	0.952***	1.082***	1.085***
	[0.946,0.957]	[0.946,0.957]	[1.071,1.094]	[1.073,1.097]
BPC(0-33)	1	1	1	1
BPC(33-66)	0.925***	0.925***	0.915***	0.916***
	[0.922,0.927]	[0.923,0.927]	[0.911,0.920]	[0.911,0.920]
BPC(66-100)	0.890***	0.890***	0.876***	0.873***
	[0.887,0.894]	[0.886,0.893]	[0.870,0.882]	[0.867,0.879]
Poverty	1.011***		1.040***	
	[1.007,1.015]		[1.033,1.048]	
Illiteracy	0.974***	0.977***	1.075***	1.085***
	[0.970,0.979]	[0.972,0.981]	[1.066,1.084]	[1.076,1.094]
Urbanization	1.022***	1.023***	0.848***	0.846***
	[1.015,1.029]	[1.016,1.029]	[0.839,0.857]	[0.837,0.855]
Fertility	0.983***	0.985***	1.055***	1.064***
	[0.979,0.987]	[0.981,0.989]	[1.047,1.062]	[1.057,1.072]
Garbage	1.026***	1.025***	0.946***	0.943***
	[1.021,1.030]	[1.020,1.030]	[0.938,0.954]	[0.935,0.950]
Physicians	1.008***	1.008***	0.940***	0.939***
	[1.005,1.011]	[1.005,1.011]	[0.935,0.946]	[0.934,0.945]
Year binaries	Yes	Yes	Yes	Yes

© 2024 Aransiola TJ et al. *JAMA Network Open.*

eTable 10 – Empirical results for mortality models with different time shocks, 2004-2019, Brazil.

	No time control	Model I (Overall)	Model II	Model III	Model IV	Model V
PBF(0-30)	1	1	1	1	1	1
PBF(30-70)	1.003	1.003	1.002	1.001	1.003	1.003
	[0.996,1.011]	[0.996,1.010]	[0.995,1.009]	[0.994,1.008]	[0.996,1.010]	[0.996,1.010]
PBF(70-100)	0.985***	0.983***	0.978***	0.978***	0.983***	0.984***
	[0.978,0.992]	[0.976,0.990]	[0.971,0.985]	[0.971,0.985]	[0.976,0.990]	[0.977,0.991]
PBF(100)	0.985***	0.982***	0.979***	0.980***	0.982***	0.981***
	[0.979,0.992]	[0.976,0.989]	[0.973,0.986]	[0.973,0.987]	[0.975,0.989]	[0.974,0.987]
FHS(0)	1	1	1	1	1	1
FHS(0-30)	0.995*	0.995*	0.995*	0.996	0.996*	0.996
	[0.990,1.001]	[0.990,1.001]	[0.990,1.000]	[0.991,1.001]	[0.990,1.001]	[0.991,1.001]
FHS(30-70)	0.934***	0.935***	0.936***	0.936***	0.938***	0.939***
	[0.929,0.939]	[0.930,0.940]	[0.931,0.941]	[0.931,0.941]	[0.933,0.943]	[0.934,0.944]
FHS(70-100)	0.950***	0.951***	0.952***	0.952***	0.956***	0.956***
	[0.944,0.955]	[0.946,0.957]	[0.946,0.958]	[0.946,0.958]	[0.950,0.961]	[0.950,0.961]
BPC(0-33)	1	1	1	1	1	1
BPC(33-66)	0.924***	0.925***	0.926***	0.927***	0.928***	0.929***
	[0.922,0.927]	[0.922,0.927]	[0.923,0.928]	[0.925,0.930]	[0.926,0.930]	[0.927,0.931]
BPC(66-100)	0.888***	0.890***	0.892***	0.893***	0.897***	0.898***
	[0.885,0.892]	[0.887,0.894]	[0.889,0.895]	[0.890,0.897]	[0.894,0.901]	[0.895,0.902]
Poverty	1.010***	1.011***	1.007***	1.008***	1.009***	1.009***
	[1.007,1.014]	[1.007,1.015]	[1.003,1.011]	[1.004,1.012]	[1.006,1.013]	[1.005,1.013]
Illiteracy	0.975***	0.974***	0.974***	0.975***	0.972***	0.970***
	[0.971,0.980]	[0.970,0.979]	[0.970,0.979]	[0.970,0.979]	[0.967,0.976]	[0.965,0.975]

© 2024 Aransiola TJ et al. *JAMA Network Open.*

Urbanization	1.021***	1.022***	1.023***	1.023***	1.025***	1.027***
	[1.014,1.028]	[1.015,1.029]	[1.016,1.029]	[1.016,1.029]	[1.018,1.031]	[1.020,1.033]
Fertility	0.984***	0.983***	0.983***	0.982***	0.980***	0.979***
	[0.980,0.988]	[0.979,0.987]	[0.980,0.987]	[0.978,0.986]	[0.976,0.984]	[0.975,0.983]
Garbage	1.025***	1.026***	1.026***	1.027***	1.029***	1.029***
	[1.020,1.030]	[1.021,1.030]	[1.022,1.031]	[1.022,1.031]	[1.024,1.033]	[1.025,1.034]
Physicians	1.008***	1.008***	1.009***	1.008***	1.010***	1.010***
	[1.005,1.011]	[1.005,1.011]	[1.006,1.012]	[1.005,1.012]	[1.007,1.013]	[1.007,1.013]
y2008		1.017***				
		[1.014,1.020]				
y2013		1.003**				
		[1.001,1.006]				
y2015		0.999				
		[0.996,1.002]				
y2006			1.019***			
			[1.016,1.022]			
y2009			1.012***		1.007***	
			[1.009,1.016]		[1.004,1.010]	
y2012			1.007***			
			[1.004,1.010]			
y2005				1.013***		
				[1.010,1.016]		
y2007				1.015***		
				[1.012,1.018]		
y2010				1.016***		
				[1.013,1.019]		
y2013					0.988***	
					[0.985,0.991]	

© 2024 Aransiola TJ et al. *JAMA Network Open.*

y2018					0.950***	
					[0.947,0.953]	
y2019						0.942***
						[0.940,0.945]
y2016						0.993***
						[0.990,0.996]
y2013						0.997**
						[0.994,1.000]
Constant	0.00447***	0.00448***	0.00449***	0.00448***	0.00457***	0.00460***
	[0.00438,0.00455]	[0.00439,0.00456]	[0.00440,0.00457]	[0.00439,0.00457]	[0.00448,0.00466]	[0.00451,0.00469]

© 2024 Aransiola TJ et al. *JAMA Network Open.*

eTable 11 – Empirical results for mortality and hospitalization models estimated using Negative Binomial and Poisson regression, 2004-2019, Brazil.

	Mortality		Hospitalization	
	Negative Binomial	Poisson	Negative Binomial	Poisson
PBF(0-30)	1	1	1	1
PBF(30-70)	0.978***	0.979**	0.976***	0.972
	[0.970,0.987]	[0.960,0.998]	[0.963,0.989]	[0.938,1.006]
PBF(70-100)	0.973***	0.972*	0.944***	0.942***
	[0.965,0.981]	[0.943,1.003]	[0.932,0.956]	[0.906,0.980]
PBF(100)	0.949***	0.948***	0.909***	0.902***
	[0.942,0.957]	[0.934,0.961]	[0.898,0.920]	[0.870,0.934]
FHS(0)	1	1	1	1
FHS(0-30)	1.000	1.002	0.963***	0.969*
	[0.992,1.007]	[0.984,1.020]	[0.953,0.974]	[0.939,1.001]
FHS(30-70)	0.933***	0.935***	0.962***	0.957***
	[0.926,0.940]	[0.921,0.950]	[0.951,0.972]	[0.928,0.987]
FHS(70-100)	0.933***	0.929***	0.989*	0.958***
	[0.926,0.940]	[0.914,0.943]	[0.978,1.001]	[0.928,0.989]
BPC(0-33)	1	1	1	1
BPC(33-66)	0.935***	0.936***	0.964***	0.978**
	[0.932,0.938]	[0.924,0.947]	[0.959,0.969]	[0.960,0.997]
BPC(66-100)	0.915***	0.915***	0.882***	0.908***
	[0.911,0.920]	[0.896,0.934]	[0.876,0.889]	[0.875,0.943]
Poverty	1.020***	1.020***	1.036***	1.046***
	[1.016,1.023]	[1.009,1.031]	[1.030,1.042]	[1.026,1.068]
Illiteracy	1.016***	1.014*	1.030***	1.027**
	[1.010,1.021]	[0.999,1.029]	[1.022,1.038]	[1.001,1.053]
Urbanization	0.984***	0.995	0.952***	1.002
	[0.977,0.992]	[0.981,1.009]	[0.941,0.962]	[0.969,1.037]
Fertility	1.013***	1.012**	1.008**	1.004
	[1.009,1.018]	[1.002,1.022]	[1.001,1.015]	[0.982,1.026]
Garbage	1.001	1.004	0.975***	0.985
	[0.995,1.006]	[0.991,1.016]	[0.968,0.983]	[0.960,1.010]
Physicians	1.005**	1.008**	0.992**	1.010*
	[1.000,1.009]	[1.001,1.015]	[0.986,0.999]	[0.999,1.022]
Year binaries	Yes	Yes	Yes	Yes
Log-likelihood	-146030.7	-150759.6	-252796.8	-1010484.4
AIC	292097.4	301553.3	505629.6	2021002.8
BIC	292252.4	301699.7	505784.7	2021149.3

eTable 12 – Empirical results for mortality and hospitalization with control for synergies between the PBF, FHS, and BPC, 2004-2019, Brazil.

	Mortality		Hospitalization	
	Without controls	With controls	Without controls	With controls
PBF(0-30)	1	1	1	1
PBF(30-70)	0.978***	0.976***	0.976***	0.973***
	[0.970,0.987]	[0.968,0.985]	[0.963,0.989]	[0.961,0.986]
PBF(70-100)	0.973***	0.967***	0.944***	0.940***
	[0.965,0.981]	[0.959,0.975]	[0.932,0.956]	[0.928,0.952]
PBF(100)	0.949***	0.955***	0.909***	0.923***
	[0.942,0.957]	[0.947,0.963]	[0.898,0.920]	[0.911,0.934]
FHS(0)	1	1	1	1
FHS(0-30)	1.000	0.993*	0.963***	0.956***
	[0.992,1.007]	[0.986,1.000]	[0.953,0.974]	[0.946,0.967]
FHS(30-70)	0.933***	0.947***	0.962***	0.966***
	[0.926,0.940]	[0.940,0.954]	[0.951,0.972]	[0.955,0.976]
FHS(70-100)	0.933***	0.943***	0.989*	0.987**
	[0.926,0.940]	[0.936,0.951]	[0.978,1.001]	[0.975,0.999]
BPC(0-33)	1	1	1	1
BPC(33-66)	0.935***	0.946***	0.964***	0.967***
	[0.932,0.938]	[0.942,0.949]	[0.959,0.969]	[0.962,0.972]
BPC(66-100)	0.915***	0.935***	0.882***	0.891***
	[0.911,0.920]	[0.930,0.940]	[0.876,0.889]	[0.884,0.899]
Poverty	1.020***	1.016***	1.036***	1.034***
	[1.016,1.023]	[1.012,1.020]	[1.030,1.042]	[1.028,1.040]
Illiteracy	1.016***	1.015***	1.030***	1.029***
	[1.010,1.021]	[1.009,1.020]	[1.022,1.038]	[1.021,1.036]
Urbanization	0.984***	0.983***	0.952***	0.953***
	[0.977,0.992]	[0.976,0.991]	[0.941,0.962]	[0.943,0.964]
Fertility	1.013***	1.015***	1.008**	1.008**
	[1.009,1.018]	[1.010,1.019]	[1.001,1.015]	[1.001,1.014]
Garbage	1.001	0.999	0.975***	0.977***
	[0.995,1.006]	[0.994,1.004]	[0.968,0.983]	[0.970,0.985]
Physicians	1.005**	1.004**	0.992**	0.993**
	[1.000,1.009]	[1.000,1.009]	[0.986,0.999]	[0.986,0.999]
PBF x FHS		1.023***		0.987**
		[1.014,1.032]		[0.975,0.999]
PBF x BPC		0.980***		0.960***
		[0.973,0.986]		[0.950,0.969]
BPCxFHS		1.055***		0.996
		[1.048,1.063]		[0.986,1.006]
Year binaries	Yes	Yes	Yes	Yes

© 2024 Aransiola TJ et al. *JAMA Network Open.*

eTable 13 - Rate Ratios from the fixed effect negative binomial models by age group for the association between mortality rates and Bolsa Família Program (BFP), Family Health Strategy (FHS), and Benefício de Prestação Continuada (BPC) coverage. The last column represents the Rate Ratios from the fixed effect negative binomial models with age-standardized mortality rate for Accidents (ICD-10:V01-X59), used as control.

	Under 5	5 - 29	30 - 69	Above 70	Accidents
BFP					
Low (0-30)	1	1	1	1	1
Intermediate (30-70)	1.014	0.993	0.970***	0.976***	0.998
	[0.984,1.044]	[0.965,1.022]	[0.959,0.982]	[0.965,0.987]	[0.675,1.477]
High (70-100)	0.957***	0.999	0.967***	0.973***	0.874
	[0.930,0.986]	[0.971,1.028]	[0.956,0.978]	[0.963,0.984]	[0.589,1.299]
Consolidated (100)	0.871***	1.016	0.939***	0.959***	0.776
	[0.847,0.895]	[0.990,1.044]	[0.929,0.949]	[0.949,0.969]	[0.538,1.120]
FHS					
Low (0)	1	1	1	1	1
Intermediate (0-30)	1.009	0.989	1.007	0.997	1.010
	[0.980,1.039]	[0.963,1.015]	[0.998,1.017]	[0.988,1.006]	[0.752,1.356]
High (30-70)	0.946***	0.942***	0.925***	0.936***	0.958
	[0.919,0.974]	[0.917,0.967]	[0.916,0.934]	[0.927,0.944]	[0.725,1.267]
Consolidated (70-100)	0.903***	0.941***	0.932***	0.933***	0.784
	[0.875,0.932]	[0.915,0.968]	[0.922,0.942]	[0.924,0.942]	[0.590,1.042]
BPC					
Low (0-33)	1	1	1	1	1
Intermediate (33-66)	0.929***	0.897***	0.929***	0.941***	0.906
	[0.917,0.941]	[0.886,0.907]	[0.925,0.933]	[0.937,0.945]	[0.777,1.056]
Consolidated (66-100)	0.840***	0.961***	0.895***	0.923***	0.907
	[0.824,0.856]	[0.945,0.977]	[0.889,0.901]	[0.918,0.929]	[0.760,1.081]
Poverty	1.048***	0.962***	1.030***	1.018***	1.209**
	[1.033,1.063]	[0.950,0.973]	[1.025,1.035]	[1.013,1.023]	[1.022,1.430]
Illiteracy	1.054***	1.055***	1.021***	1.016***	0.997
	[1.034,1.075]	[1.037,1.073]	[1.014,1.028]	[1.009,1.023]	[0.826,1.204]
Urbanization	0.931***	0.939***	0.989**	0.990*	1.053
	[0.906,0.956]	[0.917,0.962]	[0.978,0.999]	[0.980,1.000]	[0.858,1.292]
Fertility	1.073***	0.983**	1.025***	1.009***	1.108
	[1.055,1.091]	[0.969,0.998]	[1.019,1.031]	[1.003,1.015]	[0.922,1.331]
Garbage	0.955***	1.008	1.001	0.995	1.055
	[0.937,0.975]	[0.990,1.026]	[0.994,1.008]	[0.988,1.002]	[0.872,1.277]
Physicians	0.979***	1.002	1.001	1.006**	1.123
	[0.963,0.995]	[0.987,1.016]	[0.995,1.006]	[1.000,1.011]	[0.968,1.303]
Years	Yes	Yes	Yes	Yes	Yes

Note: Rate Ratio (RR) coefficients; 95% confidence intervals in parentheses; Time shocks are controls for specific years of economic crisis -- 2008, 2013, and 2015; ***, ** and * denote significance at 1%, 5%, and 10% respectively. The total number of observations is 40.762 -- 2.548 municipalities and 16 years (from 2004 to 2019).

© 2024 Aransiola TJ et al. *JAMA Network Open.*

4.3. Additional outcomes

In eTable 14, we present the models for hospitalization by age group similar to that presented for mortality in Table 3 of the manuscript. In eTable 15, we present the overall models for mortality and hospitalization where we decompose the coverage of the BPC into deficiency and old-age coverage to verify which of both coverages is mostly responsible for the high magnitude of effect found for the BPC.

eTable 14 – Empirical results for hospitalization models by age group, 2004-2019, Brazil.

Variables	Under 5	5 - 29	30 - 69	Above 70
PBF(0-30)	1	1	1	1
PBF(30-70)	1.040***	0.995	0.955***	0.928***
	[1.018,1.063]	[0.981,1.008]	[0.942,0.969]	[0.911,0.945]
PBF(70-100)	1.038***	0.958***	0.919***	0.912***
	[1.017,1.061]	[0.945,0.971]	[0.907,0.932]	[0.896,0.928]
PBF(100)	1.027***	0.917***	0.883***	0.876***
	[1.007,1.048]	[0.906,0.928]	[0.872,0.895]	[0.862,0.891]
FHS(0)	1	1	1	1
FHS(0-30)	0.890***	0.966***	0.970***	0.988
	[0.874,0.906]	[0.954,0.977]	[0.959,0.981]	[0.974,1.003]
FHS(30-70)	0.928***	0.975***	0.949***	0.931***
	[0.911,0.945]	[0.964,0.986]	[0.938,0.960]	[0.918,0.945]
FHS(70-100)	0.960***	1.002	0.999	0.957***
	[0.942,0.979]	[0.990,1.015]	[0.986,1.011]	[0.943,0.972]
BPC(0-33)	1	1	1	1
BPC(33-66)	0.977***	0.981***	0.944***	0.915***
	[0.969,0.985]	[0.975,0.986]	[0.939,0.949]	[0.909,0.921]
BPC(66-100)	0.868***	0.910***	0.855***	0.831***
	[0.858,0.879]	[0.903,0.918]	[0.848,0.862]	[0.823,0.840]
Poverty	1.042***	1.030***	1.037***	1.027***
	[1.032,1.052]	[1.024,1.036]	[1.031,1.044]	[1.019,1.036]
Illiteracy	1.085***	1.039***	1.033***	1.028***
	[1.072,1.099]	[1.031,1.048]	[1.025,1.042]	[1.017,1.039]
Urbanization	0.865***	0.959***	0.935***	0.932***
	[0.851,0.880]	[0.948,0.970]	[0.924,0.946]	[0.918,0.945]
Fertility	1.038***	1.014***	1.009**	1.024***
	[1.027,1.050]	[1.007,1.021]	[1.001,1.016]	[1.014,1.034]
Garbage	0.958***	0.972***	0.982***	0.942***
	[0.946,0.970]	[0.964,0.980]	[0.973,0.990]	[0.932,0.952]
Physicians	0.956***	0.993**	0.985***	0.980***
	[0.946,0.966]	[0.986,0.999]	[0.978,0.991]	[0.972,0.988]
Year binaries	Yes	Yes	Yes	Yes

eTable 15 – Empirical results for mortality and hospitalization models with decomposed BPC coverage, 2004-2019, Brazil.

	Mortality		Hospitalization	
	Without controls	With controls	Without controls	With controls
PBF(0-30)	1	1	1	1
PBF(30-70)	0.977***	0.976***	0.977***	0.977***
	[0.968,0.985]	[0.967,0.984]	[0.965,0.990]	[0.964,0.989]
PBF(70-100)	0.968***	0.970***	0.940***	0.945***
	[0.960,0.976]	[0.962,0.978]	[0.928,0.952]	[0.933,0.957]
PBF(100)	0.943***	0.948***	0.909***	0.919***
	[0.935,0.950]	[0.941,0.956]	[0.899,0.920]	[0.908,0.930]
FHS(0)	1	1	1	1
FHS(0-30)	1.003	1.005	0.963***	0.968***
	[0.995,1.010]	[0.998,1.013]	[0.952,0.973]	[0.958,0.979]
FHS(30-70)	0.933***	0.934***	0.969***	0.971***
	[0.927,0.940]	[0.928,0.941]	[0.958,0.979]	[0.961,0.982]
FHS(70-100)	0.930***	0.933***	0.995	1.000
	[0.922,0.937]	[0.926,0.941]	[0.984,1.007]	[0.989,1.012]
BPC(0-33) - Deficiency	1	1	1	1
BPC(33-66) - Deficiency	0.934***	0.935***	0.961***	0.964***
	[0.930,0.937]	[0.932,0.938]	[0.957,0.966]	[0.959,0.969]
BPC(66-100) - Deficiency	0.905***	0.915***	0.863***	0.882***
	[0.901,0.910]	[0.911,0.920]	[0.857,0.870]	[0.876,0.889]
BPC(0-33) - Elderly	1	1	1	1
BPC(33-66) - Elderly	0.934***	0.935***	0.961***	0.964***
	[0.930,0.937]	[0.932,0.938]	[0.957,0.966]	[0.959,0.969]
BPC(66-100) - Elderly	0.905***	0.915***	0.863***	0.882***
	[0.901,0.910]	[0.911,0.920]	[0.857,0.870]	[0.876,0.889]
Poverty		1.017***		1.027***
		[1.013,1.020]		[1.021,1.033]
Illiteracy		1.012***		1.028***
		[1.007,1.018]		[1.020,1.036]
Urbanization		0.984***		0.953***
		[0.977,0.992]		[0.942,0.963]
Fertility		1.014***		1.006*
		[1.010,1.019]		[0.999,1.012]
Garbage		1.001		0.981***
		[0.996,1.006]		[0.973,0.988]
Physicians		1.004**		0.993**
		[1.000,1.009]		[0.987,0.999]
Year binaries	Yes	Yes	Yes	Yes

© 2024 Aransiola TJ et al. *JAMA Network Open.*

Note: Incidence-Rate Ratio (IRR) coefficients; Confidence intervals in parentheses; Time shocks are controls for specific years of economic crisis -- 2008, 2013, and 2015; ***, ** and * denote significance at 1%, 5%, and 10% respectively. The total number of observations is 40.762 -- 2.548 municipalities and 16 years (from 2004 to 2019).

eTable 16 – Spacial Model ICAR and CAR, 2004-2019, Brazil.

**Variable**	**ICAR**	**CAR**
PBF(0-30)	1	1
PBF(30-70)	0.984***(0.973,0.995)	0.984***(0.973,0.995)
PBF(70-100)	0.952***(0.941,0.963)	0.952***(0.941,0.963)
PBF(100)	0.937***(0.927,0.947)	0.937***(0.927,0.948)
ESF(0)	1	1 (1,1)
ESF(0-30)	0.991 (0.982,1.000)	0.990***(0.982,0.999)
ESF(30-70)	0.944***(0.937,0.952)	0.944***(0.936,0.951)
ESF(70-100)	0.927***(0.920,0.934)	0.927***(0.919,0.934)
BPC(0-33)	1	1
BPC(33-66)	0.966***(0.961,0.970)	0.966***(0.961,0.970)
BPC(66-100)	0.949***(0.943,0.955)	0.950***(0.944,0.956)
Poverty	1.025***(1.019,1.031)	1.026***(1.02,1.032)
Illiteracy	1.004 (0.998,1.011)	1.006 (0.999,1.012)
Urbanization	1.033***(1.026,1.04)	1.034***(1.027,1.041)
Fertility	1.010***(1.005,1.015)	1.010***(1.005,1.016)
Garbage	1.020***(1.014,1.026)	1.020***(1.015,1.026)
Physicians	1.007***(1.003,1.011)	1.006***(1.002,1.01)
y2008	1.022***(1.016,1.027)	1.022***(1.016,1.027)
y2013	1 (0.994,1.005)	1 (0.994,1.005)
y2015	0.992***(0.987,0.998)	0.992***(0.987,0.998)

Note: ICAR: Intrinsic Conditional Autorregressive Correlation. CAR: Conditional Autorregresive Correlation. Neighbors based correlations functions (Random spatial effect).

© 2024 Aransiola TJ et al. *JAMA Network Open.*

5. Deaths averted by BFP, FHS, and BPC programs during 2004-19

To simulate deaths avoided due to BFP, FHS, and BPC programs in 2004-19 period, we predicted coefficient $E(Y_{it} \mid X)$, here X represents the set of covariates including the interventions, and Y_{it} are the mortality rate at municipality i, in year t. Thus, the Monte Carlo methodology was used to get more accurate results compared with conventional methods such as the use of the normal distribution. It can be summarized in the following steps:

1. Predict the intervention values for the retrospective period (2004-2019) initially using the same coefficients as the main model (Table 2 and 3).
2. Simulate a new Y_{it} from the negative binomial distribution using the estimated parameters from the retrospective study, changing only the BFP, FHS, and BPC coefficients to rescue the baseline (0% coverage) and compared with real deaths, making the difference between them, and adding them up over the different years.
3. Get the predictions $E(Y_{it} \mid X)$ using the new simulated variable Y_{it}, here X represents the set of covariates including the interventions.
4. Get back to step 1.

The algorithm ended when the number of desired Monte Carlo simulations M is reached. For each outcome, 10,000 simulations were performed, chosen based on the stabilization of the estimates.

The eTable 17 show this simulation, in which BFP, FHS, and BPC programs avoiding 1,462,626 (95% CI: 1,332,128-1,596,924) overall deaths and 177,101 (95% CI: 167,664-186,836) child deaths between 2004-19 in the hypothetical case these programs did not exist (0% coverage).

eTable 17 –Death avoided by BFP, FHS, and BPC programs coverage during 2004-19.

	**Averted deaths until 2019**		
Age Group	Estimate	UL	LL
Overall	1,462,626	1,332,128	1,596,924

Note: BFP=Bolsa Família Program, FHS=Family Health Strategy, BPC=Benefício de Prestação Continuada, UL=Upper limit (95% CI), LL=Lower limit (95% CI).

© 2024 Aransiola TJ et al. *JAMA Network Open.*

eAppendix 3. FORECASTING ANALYSIS

6. Description of the forecasting methodology

The following section provides details of the forecasting process in accordance with standard international modeling reporting guidelines (ISPOR-SMDM). The modeling approach adopted for this study was developed based on two stages.

In the first stage, a synthetic cohort of all Brazilian municipalities for the period 2020-2030 was created as an extension of a longitudinal dataset of 5507 municipalities for 2000-2019 obtained from the sources detailed in eTable 1. Simulated municipality-specific trends for poverty rates and the other demographic and socioeconomic variables were obtained according to economic crisis scenarios for the years 2020-2030. BFP, FHS and BPC coverage were simulated according to social protection policy response scenarios options.

In the second stage, for each year and each municipality, the mortality rate for all the municipalities was estimated as the outcome of the same multivariate fixed effects regressions, using the forecast demographic, socioeconomic and exposure variables (BFP and FHS coverage) as input values.

7. Purpose of the forecasting and its applications

The developed model had the overall purpose to simulate the effects of socioeconomic and policy coverage changes on health outcomes in Brazil using ecologic-level data and - when available - retrospective ecologic datasets. Elements of flexibility have been introduced in the code to allow simulation of different sets of variables and different regression models.

8. Inputs, outputs, and other parameters

8.1. Scenarios of poverty and coverage of social welfare programs

In order to develop forecasting, exponential functions were used to simulate the covariates behavior for the next 11 years (2020-2030). Regarding the poverty rate, an increasing scenario was considered for the first years (economic crisis period). This is described by the equation,

$$x_t = x_{2019} + c_1 x_{2019}(1 - \exp(-k_1 t)), \quad (2)$$

for the remaining years, we consider the exponential decay,

$$x_t = x_{2019} - c_2 x_{2019}(1 - \exp(-k_2 t)), \quad (3)$$

where the parameters c_1, k_1, c_2, k_2 were settled according to different available sources.

With respect to the intervention variables (BFP, FHS, BPC) under the mitigation scenario, they were considered as having the same behavior of the poverty rate (mitigation effect) during the economic crisis[5]. For the post - crisis, the interventions were simulated using the exponential

© 2024 Aransiola TJ et al. *JAMA Network Open.*

decay in equation (3). The decreasing rate k2 in this case, was settled as half the poverty to simulate the transition period between the crisis and recovery scenarios.

Under the austerity scenarios, the interventions xt, austerity were considered to follow an exponential decay which shows directly the percentage of decrease per year, this allowed to support the policies simulation according to situations that concerns the Brazilian government expenditure.[24] The equation below describes this dynamic.

$$x_{t,aust} = x_{t,aust}\,(1-p)^t$$

where p is the percentage of decreasing for each intervention and t refers to the year.

We simulated three economic crisis scenarios using the increase in the poverty rates, which was calculated using the microdata from the Brazilian National Household surveys from (PNAD) 2004 to 2019, and the special PNAD conducted during the COVID-19 pandemic for the year 2020. Poverty is defined as the percentage of households below the national poverty line, which is also used as an eligibility condition for the Bolsa Familia Conditional Cash Transfer program. The magnitude of the economic crisis is represented by the percentage variation of the poverty rate from 2019 to 2020 without considering the COVID Emergency cash transfer (*Auxílio Emergencial*), hence the COVID10-related economic crisis. Recent reports show that the acute increase of poverty rate from 2020 to 2021 (about 22,7%) is significantly higher compared to the annual poverty increase used as economic crises scenarios in this study.[25]

The economic crisis scenarios considered in this analysis were simulated as follows:

- Shorter Economic Crisis scenario: A milder and shorter economic crisis, with an increase in poverty rates for the first three years (2020 - 2022). This behavior was generated using equation (2), with parameters c1= 0.6, k1 =0.1 . On the other side, For the post-crisis period (2023 -2030), poverty rates were simulated by using equation (3) with parameters c2 = 0.53 e k2=0.2 .
- Medium Economic Crisis scenario: A medium economic crisis with a larger increase in the poverty rate for the first 5 years (2020-2024). This behavior was generated using equation (2), with parameters c1= 1.2, k1 =0.2 . As in the first scenario, For the post-crisis period (2025 -2030), the poverty rates were simulated by using equation (3) with parameters c2 = 0.53 e k2=0.2.
- Longer Economic Crisis scenario: A longer economic crisis was created using similar parameters as the Medium Economic Crisis scenarios, but with an increase sustained over 7 years (from 2015 to 2021).

In response to the economic crisis, three policy responses were considered in the main analysis:

- Mitigation scenario: a mitigation strategy with a proportional behavior of the BFP, FHS, and BPC to the poverty scenarios, during the corresponding simulated economic crisis. In this case, these interventions were generated in the same way as poverty rates, considering the same equation and parameters according to each period and scenario.
- Baseline scenario: derived from a validated model - already employed in previous studies[5,6] - that projected the effects of the current fiscal austerity measures due to the *Emenda Constitucional* 95 (EC95) on the coverage of the three interventions. This scenario was simulated according to the equation (4) considering a percentage of decrease of 5.0%, as in previous studies.[5,6]

- Severe Austerity scenario: based on the reduction of BFP, FHS, and BPC proportional to the reduction of government expenditure on social protection (excluding cash transfer programs) observed from 2014-2019 24. This scenario was simulated according to the equation (4) considering a percentage of decrease of 9.8%. This percentage was derived from the reduction of government expenditure on social protection (excluding cash transfer programs) observed from 2014-2019.[24]

The Figures 16 - 19 shows the behavior of the poverty scenarios and the interventions according to mitigation and austerity policies.

Regarding the control covariates, they were simulated by using the equations (2) and (3) depending on their trend (increasing or decreasing). The figure 20 shows the behavior of each one for 5507 municipalities.

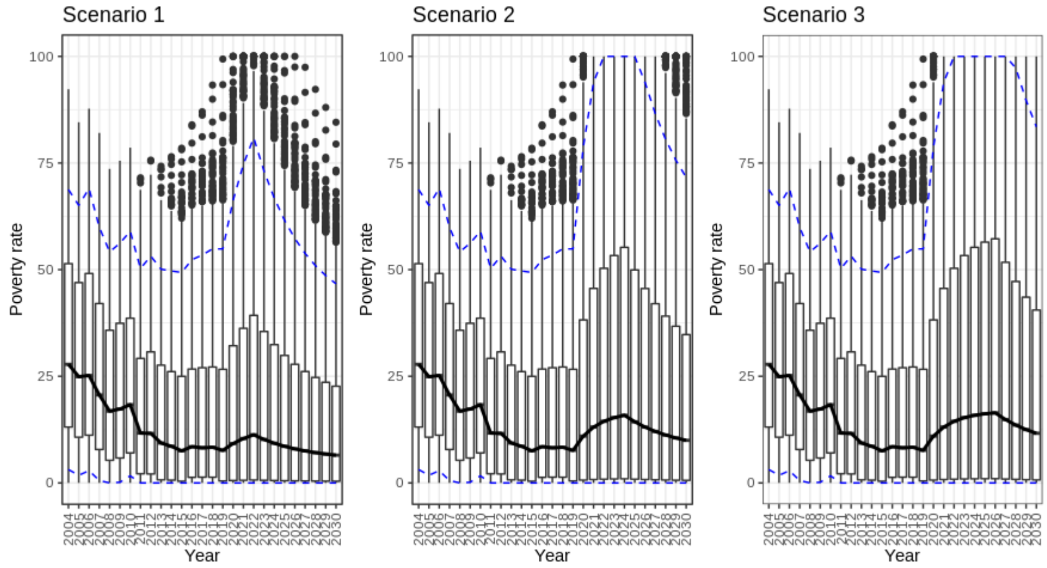

eFigure 16. Forecasted poverty rate.

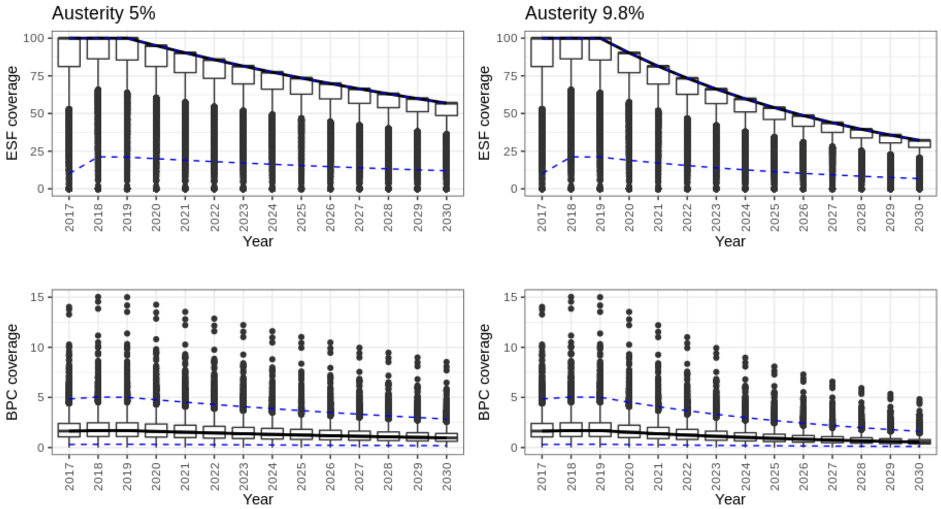

eFigure 17. Simulated FHS and BPC coverage under the austerity scenarios.

© 2024 Aransiola TJ et al. *JAMA Network Open.*

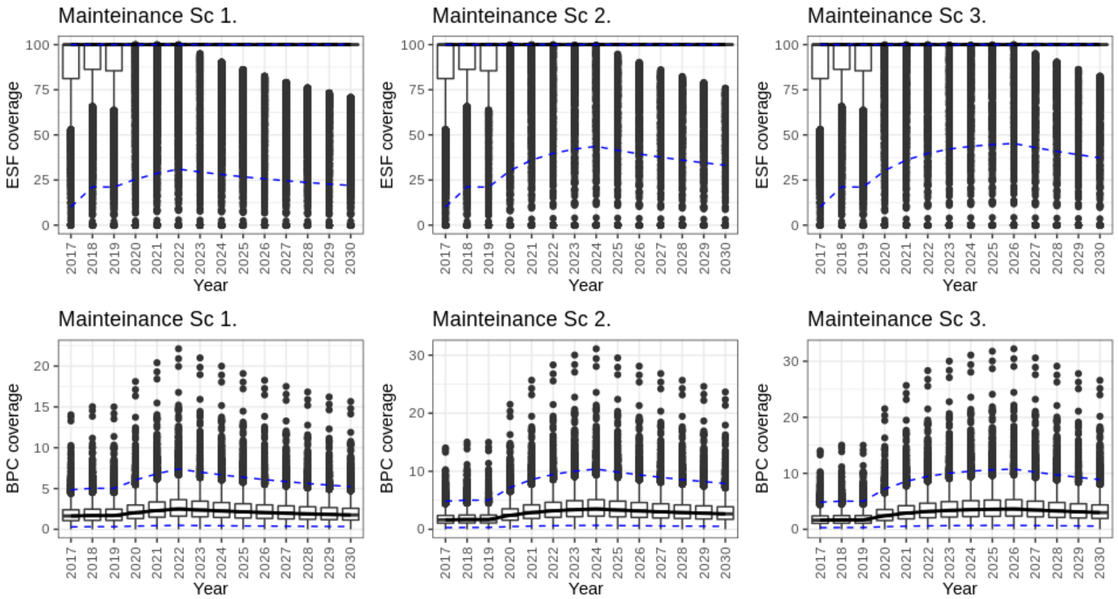

eFigure 18. Simulated FHS and BPC coverage under the mitigation scenarios.

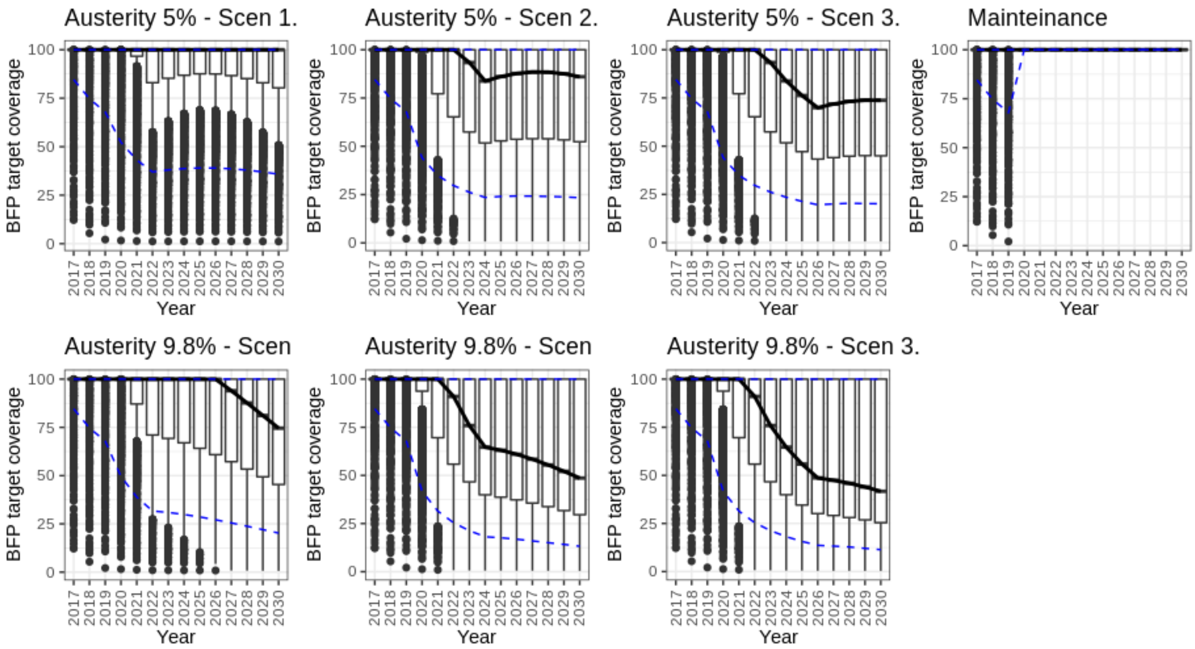

eFigure 19. Simulated BFP coverage according to different policies and poverty scenarios

© 2024 Aransiola TJ et al. *JAMA Network Open.*

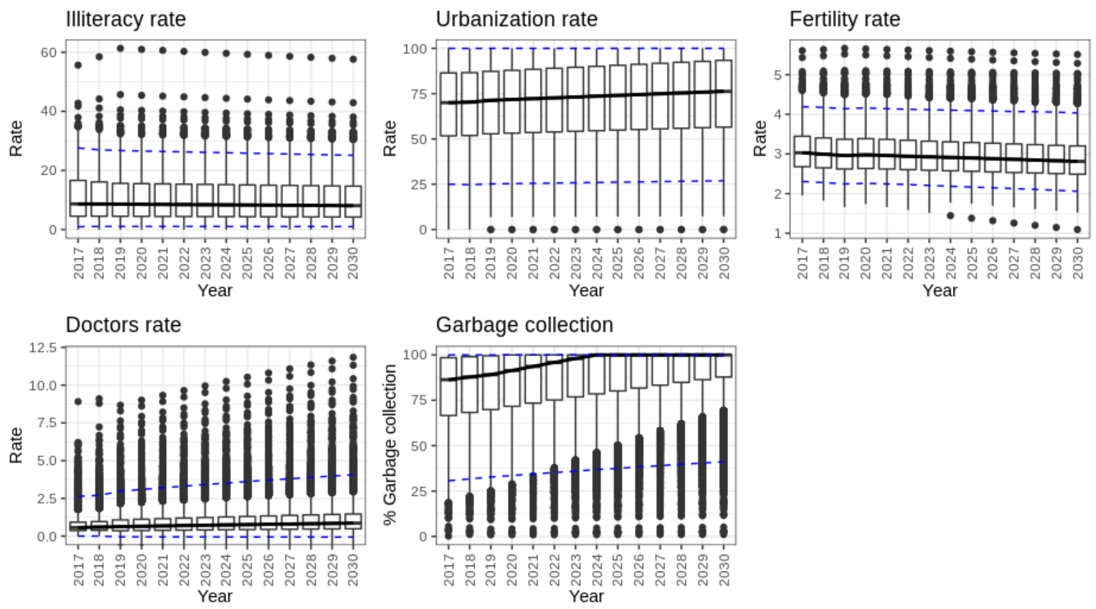

eFigure 20. Forecasted covariates.

9. Prediction methodology

To generate predictions and confidence intervals for each response Y_{it}, the Monte Carlo methodology was used. This procedure allows to get more accurate results compared with conventional methods such as the use of the normal distribution. It can be summarized in the following steps

5. Simulate the intervention values for the forecasting period (2020 -2030) using the mitigation and austerity scenarios settled in previous sections. Also simulate the control covariates using equations (2) and (3) and following their trend.
6. Simulate a new Y_{it} from the negative binomial distribution using the estimated parameters from the retrospective study and the forecasted covariates.
7. Get the predictions $E(Y_{it} \mid X)$ using the new simulated variable Y_{it}, here X represents the set of covariates including the interventions.
8. Get back to step 1.

The algorithm ended when the number of desired Monte Carlo simulations M is reached. The predictions and confidence interval estimated for Y_{it} will be the mean and the percentiles 2.5% and 97.5% of the M simulations respectively. For each outcome and each scenario, 10,000 simulations were performed, allowing parameter values to vary in each simulation cycle according to their assumed underlying distribution. The number 10,000 was chosen based on the stabilization of the estimates.

8.1 Calibration of the models

To calibrate the models for the 5,507 Brazilian municipalities, we ran the simulation for the period 2004-2019 calibrating the fixed effects term of the municipalities, and the time fixed effects of the regression, through the comparison of the simulated average municipal mortality rates of all

municipalities with the real average municipal mortality rates of Brazil for the same period. The sum of squared errors (SSE) was used as measure of goodness of fit.

8.2. Internal validation of each model

Internal validity of the model was assessed fitting the fixed effects negative binomial multivariate regression described above - and used for the microsimulation - on the synthetic dataset created for the period 2020-2030, and verifying that the obtained coefficients for each variable were the same than the ones introduced as inputs in the model (and derived from the retrospective impact evaluation).

8.3. External validation of each model

The external validation of the model was undertaken comparing the overall national mortality rate (computed for each municipality) forecasted using microsimulations, with the official Brazilian mortality estimates (overall) during the years 2010-2019, which are the most up-to-date available, and estimating the linear regression and the correlation coefficients (R2) of predicted vs observed values, as shown in eFigure 21.

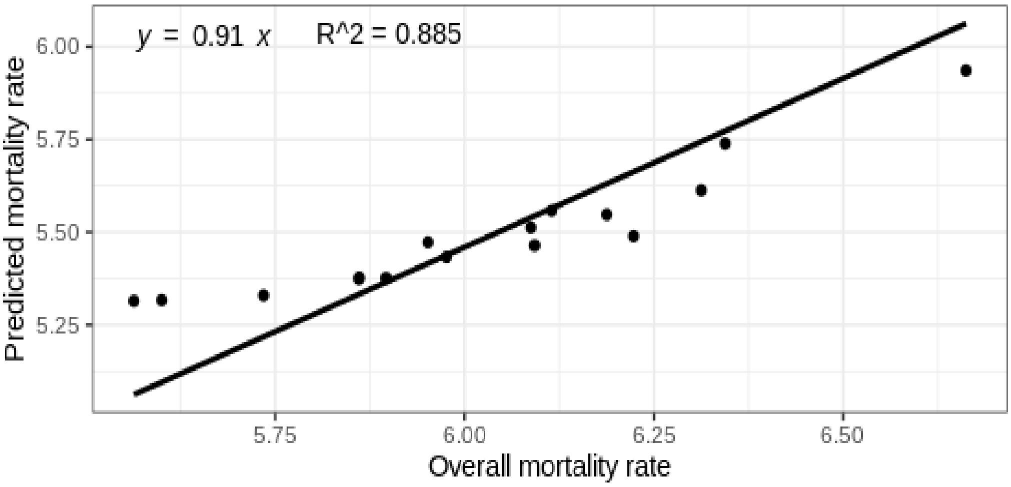

eFigure 21. Linear regression and correlation coefficient (R2) of predicted vs observed values, and trend of the simulated overall mortality rate vs the official Brazilian mortality rate estimates for the period 2010-2019.

10. Summary of results

Tables and Figures illustrating the results are available in the main manuscript.

In the mitigation scenario, mortality rates are decreasing over the next decade; in the baseline austerity, mortality rates are slightly increasing; and; in the strong austerity scenario, mortality rates increase expressively at the end of the period. In 2030, for overall mortality, the RR between mitigation and baseline would be 0.934 (95% CI: 0.916 - 0.951), whereas the RR between the mitigation and severe austerity is 0.878 (95% CI:0.859-0.905). This corresponds to 778,636 (95%CI: 664,955- 893,433) averted deaths from 2020-30 in the case of mitigation versus baseline, and 1,424,215 (95%CI: 1,264,187 - 1,587,362) averted deaths for mitigation versus strong austerity. For under-5 mortality, the first comparison corresponds to a RR and averted deaths of 0.703 (95%CI: 0.678-0.727) and 127,916 (95%CI: 120,315– 135,545), respectively, whereas the

© 2024 Aransiola TJ et al. *JAMA Network Open.*

second comparison corresponds to a RR and averted death of 0.601 (95%CI: 0.573-0.625) and 205,234 (95%CI: 194,140 – 216,243), respectively. In the web appendix, eTable 12, the RR and averted deaths are reported according to alternative firmer or softer economic crisis scenarios, showing results of similar magnitude.

9.1. Sensitivity analysis

To evaluate how a lengthening of the economic crisis could affect mortality rates we additionally modelled the impact of austerity and social protection mitigation considering two additional poverty scenarios (see Figures 16, 17 and 18). eTables 18 and 19 are consistent with the results found for the poverty scenario 2 (see the main manuscript) showing that the averted deaths decrease as the policies conditions improve in terms of increase the coverage of the different social programs

eTable 18: Rate ratio (RR) in 2030 and cumulative difference in overall deaths over the period 2020-2030 between alternative policy scenarios, according to different the Economic Crisis Scenario.

	Mitigation vs Baseline		Mitigation vs Austerity	
Year	Rate Ratio (number)	[95% Prediction Intervals]	Rate Ratio (number)	[95% Prediction Intervals]
Shorter Economic Crisis				
Overall mortality	0.948	[0.931 - 0.965]	0.892	[0.873- 0.909]
Avoidable deaths	(660,661)	[571,301 – 750,621]	(1,305,359)	[1,163,659 – 1,449,256]
Medium Economic Crisis				
Overall mortality	0.934	[0.916 - 0.951]	0.878	[0.859 - 0.905]
Avoidable deaths	(778,636)	[664,955- 893,433]	(1,424,215)	[1,264,187 - 1,587,362]
Longer Economic Crisis				
Overall mortality	0.931	[0.913- 0.948]	0.875	[0.856- 0.886]
Avoidable deaths	(787,026)	[627,553 – 950,033]	(1,430,144)	[1,243,353 – 1,627,265]

© 2024 Aransiola TJ et al. *JAMA Network Open.*

eTable 19: Rate ratio (RR) in 2030 and cumulative difference in under-5 deaths over the period 2020-2030 between alternative policy scenarios, according to different the Economic Crisis Scenario.

	Mitigation vs Baseline		Mitigation vs Austerity	
Year	Rate Ratio (number)	[95% Prediction Intervals]	Rate Ratio (number)	[95% Prediction Intervals]
Shorter Economic Crisis				
Overall mortality	0.755	[0.731- 0.780]	0.649	[0.623- 0.657]
Avoidable deaths	(105,544)	[99,510– 111,671]	(182,531)	[172,600 – 192,523]
Medium Economic Crisis				
Overall mortality	0.703	[0.678 - 0.727]	0.601	[0.573 - 0.625]
Avoidable deaths	(127,916)	[120,315- 135,545]	(205,234)	[194,140 – 216,243]
Longer Economic Crisis				
Overall mortality	0.694	[0.669-0.718]	0.592	[0.566- 0.603]
Avoidable deaths	(130,554)	[122,734– 138,453]	(208,027)	[196,744– 219,221]

eTable 20 – Avoidable hospitalization by BFP, FHS, and BPC programs coverage over the period 2020-2030 between alternative policy scenarios.

	**Avoidable hospitalization**		
	Estimate	UL	LL
Austerity (5 %)	4,697,468	4,006,945	5,412,836
Austerity (10 %)	6,593,224	5,534,591	7,651,327

Note: BFP=Bolsa Família Program, FHS=Family Health Strategy, BPC=Benefício de Prestação Continuada, UL=Upper limit (95% CI), LL=Lower limit (95% CI).

11. Main limitations

The main limitation of the study is the uncertainty around the future macroeconomic scenarios in Brazil, due to the current extremely unstable political and economic situation, which creates uncertainty around the forecasting of poverty rates, income, and the other independent variables. For that reason, several scenarios have been simulated in sensitivity analyses which produced comparative findings. Another limitation is that the modelling of austerity measures is focused on BFP, FHS and BPC as there is strong evidence that these policies confer protective effects for overall and childhood morbidity and mortality from previous studies.[1]

© 2024 Aransiola TJ et al. *JAMA Network Open.*

Our estimates of the impact of austerity measures on different age groups are probably conservative as they do not reflect constraints in other areas of public spending e.g., education, housing and other welfare programs which have known impacts on poverty and health. Moreover, austerity measures recently enshrined in the constitution of Brazil means that public spending will only increase in line with inflation, which will not account for the demographic growth of the population, its ageing processes, and growing costs associated with new healthcare treatments and technologies.[26–28] Another limitation of the study is that we do not model the impact of the increased coverage of BFP on poverty rate dynamics, assuming that poverty rates influence BFP coverage and not the contrary.

This is mainly due to the assumption that WB simulations of poverty increase during economic crisis already account for BFP effects, and because reliable parameters were not available at the moment of writing.

eAppendix 4. TRIANGULATION ANALYSES

We analyzed the effect of FHP, BFP and BPC programs on overall mortality by difference-in-difference (DID) with propensity score matching (PSM) as a triangulation approach.29 For the Bolsa Familia Program, we used 602 municipalities with low coverage (BFP<=29.9%) and compared them with 1,944 municipalities with high coverage (BFP>30%) in the years 2004 and 2019. We used the same strategy for the Family Health Program, separating municipalities with low FHP coverage (FHP<=29.9%) in 2004 (n=223) from those with high coverage (FHP>30%; n=1,827), totaling 2,050 municipalities analyzed for FHP in the years 2004 and 2019. In the case of the social pension program, we used 485 municipalities with low coverage (BPC<=0-32.9 percentile) and compared them with 1,231 municipalities with high BPC coverage (BPC>33-100 percentile) in the years 2004 and 2019. Thus, municipalities with low coverage of each of these programs receive a value equal to 0 (control) and municipalities with medium to high coverage receive a value equal to 1 (treated).

We also tested two different ways of estimating the DID: the first approach we used the "diff" command in STATA, with the Propensity Score Matching performed before the DID model. On the second approach, we used the step-by-step procedure described in the World Bank handbook,2 which allows estimating DID with a negative binomial panel of fixed effects and with coefficients in Incidence-rate ratio (IRR). We prefer this second one because it allows a more direct comparison with the results reported in the manuscript. The table below further separates these analysis strategies.

eTable 21. Different strategies for difference in difference models

Analysis strategies	Control (dummy=0)	Treated (dummy=1)	Time	Table
BFP and FHP	Low coverage (<30%)	Intermediate to high coverage (>=30%)	2004 and 2019	Table 4 (“diff”) –DID with PSM;
BPC	Low BPC coverage (<33 tercile)	Intermediate to high BPC coverage (>33 tercile)	2004 and 2019	Table 7 – DID with IRR fixed effect negative binomial models

© 2024 Aransiola TJ et al. *JAMA Network Open.*

eTables 22 and 23 show the result of DID models, and coverages of FHP, BPC and BFP programs were associated with a statistically significant reduction in child mortality rates, with incidence-rate ratios (IRR) of 0·985(95%CI:0·983-0·986), 0·993(95%CI:0·992-0·995), and 0·975(95%CI:0·975-0·976), respectively.

Even after approximating the municipalities by characteristics observed by the kernel matching method, the results are persistent for all analyzed programs, with a difference in difference showing a greater reduction in overall mortality among municipalities with high FHP, BPC and BFP coverage when compared to municipalities with low or no coverage of these programs, in the years 2004 and 2019. Thus, these tree programs contributed to the reduction of infant mortality, being compatible with the results already found and described in the main manuscript, so that these results by DID with PSM reinforce the results found by the fixed effect panel with negative binomial, being a form of triangulation of results.

eTable 22. **Difference-in-difference with Propensity Score Matching for the association between child mortality rates and intermediate to high Conditional Cash Transference (CCT) coverage, in 2004 and 2019.**

	**Overall Mortality**		
	**FHP**	**BPC**	**BFP**
Before (2004)			
Control	6.842	6.477	6.151
Treated	6.352	6.460	6.419
1st Difference (T-C)	-0.490***	-0.017	0.268***
	(0.051)		(0.096)
After (2019)			
Control	5.699	5.772	5.369
Treated	5.600	5.434	5.338
2nd Difference (T-C)	-0.099*	-0.338***	-0.031
	(0.051)	(0.091)	(0.096)
**Diff-in-Diff**	**0.391***<br>(0.073)**	**-0·321***<br>(0·110)**	**-0.299**<br>(0.136)**

Source: Author's data analysis for 5,092 observations – 2,546 municipalities in Brazil, at the years 2004 and 2019.
Note: Data are in mortality rate per 1,000 livebirths, with standard errors in parentheses. The symbols '***' , '**' and '*' denote significance at 1%, 5%, and 10% respectively. We use the STATA "diff" command, with kernel matching (PSM) to approximate the compared municipalities according to their observable characteristics.

eTable 23. Incidence-Rate Ratios from the difference-in-difference fixed effect negative binomial models by under 5 age group for the association between mortality rates and intermediate to high Conditional Cash Transference (CCT) coverage, in 2004 and 2019.

	**Overall Mortality**		
	**FHP**	**BPC**	**PBF**
**FHP coverage (dummy)**	5.58732e+14***<br>[2.367e+13-1.319e+16]	-	-
Intermediate to high (>=30%)	0.985***<br>[0.983-0.986]	-	-
**BPC coverage (dummy)**	-	1359066.3***<br>[66625.811-27722907.992]	-
Intermediate to high (>33 tercile)	-	0.993***<br>[0.992-0.995]	-
**BFP target coverage (dummy)**	-	-	3.72256e+22***<br>[1.325e+22-1.046e+23]
Intermediate to high (>=30%)	-	-	0.975***<br>[0.975-0.976]
Proportion of individuals older than 15 years who are illiterate (%)	0.996***<br>[0.993-0.999]	0.993***<br>[0.990-0.996]	0.982***<br>[0.980-0.983]
Poverty rate (%)	0.997***<br>[0.996-0.998]	0.999<br>[0.998-1.001]	1.001***<br>[1.000-1.001]

© 2024 Aransiola TJ et al. *JAMA Network Open.*

Urbanization	0.998***	1	0.999**
	[0.997-0.999]	[0.998-1.001]	[0.997-1.000]
Fertility rate	1.021	1.120***	1.002***
	[0.989-1.055]	[1.082-1.160]	[1.002-1.003]
Doctor rate	0.980***	0.931***	0.999
	[0.969-0.991]	[0.920-0.943]	[0.996-1.002]
Garbage	1.003***	1.001	1.003***
	[1.002-1.004]	[1.000-1.002]	[1.002-1.003]
**Number of observations**	**4,072**	**3,202**	**5,057**
**Number of municipalities**	2,050	1,716	2,546

Source: Author's data analysis for 5,057 observations – 2,546 municipalities in Brazil, in 2004 and 2019 period.
Note: Data are in Incidence-Rate Ratio (IRR) coefficients (95% CI) unless otherwise specified. The confidence intervals are in brackets. The symbols '***' , '**' and '*' denote significance at 1%, 5%, and 10% respectively.

© 2024 Aransiola TJ et al. *JAMA Network Open.*

eReferences

1 Rasella D, Aquino R, Santos CAT, Paes-Sousa R, Barreto ML. Effect of a conditional cash transfer programme on childhood mortality: A nationwide analysis of Brazilian municipalities. *Lancet* 2013; **382**. DOI:10.1016/S0140-6736(13)60715-1.

2 Lucas ADP, de Oliveira Ferreira M, Lucas TDP, Salari P. The intergenerational relationship between conditional cash transfers and newborn health. *BMC Public Health* 2022; **22**. DOI:10.1186/s12889-022-12565-7.

3 Guanais FC. The combined effects of the expansion of primary health care and conditional cash transfers on infant mortality in Brazil, 1998-2010. Am. J. Public Health. 2015; **105**. DOI:10.2105/AJPH.2013.301452.

4 Machado DB, Rodrigues LC, Rasella D, Barreto ML, Araya R. Conditional cash transfer programme: Impact on homicide rates and hospitalisations from violence in Brazil. *PLoS One* 2018; **13**. DOI:10.1371/journal.pone.0208925.

5 Rasella D, Basu S, Hone T, Paes-Sousa R, Ocké-Reis CO, Millett C. Child morbidity and mortality associated with alternative policy responses to the economic crisis in Brazil: A nationwide microsimulation study. *PLoS Med* 2018; **15**. DOI:10.1371/journal.pmed.1002570.

6 Rasella D, Hone T, De Souza LE, Tasca R, Basu S, Millett C. Mortality associated with alternative primary healthcare policies: A nationwide microsimulation modelling study in Brazil. *BMC Med* 2019; **17**. DOI:10.1186/s12916-019-1316-7.

7 Hone T, Rasella D, Barreto M, Atun R, Majeed A, Millett C. Large reductions in amenable mortality associated with brazil’s primary care expansion and strong health governance. *Health Aff* 2017; **36**. DOI:10.1377/hlthaff.2016.0966.

8 Rasella D, Aquino R, Barreto ML. Reducing childhood mortality from diarrhea and lower respiratory tract infections in brazil. *Pediatrics* 2010; **126**. DOI:10.1542/peds.2009-3197.

9 Rasella D, Harhay MO, Pamponet ML, Aquino R, Barreto ML. Impact of primary health care on mortality from heart and cerebrovascular diseases in Brazil: A nationwide analysis of longitudinal data. *BMJ* 2014; **349**. DOI:10.1136/bmj.g4014.

10 Hone T, Rasella D, Barreto ML, Majeed A, Millett C. Association between expansion of primary healthcare and racial inequalities in mortality amenable to primary care in Brazil: A national longitudinal analysis. *PLoS Med* 2017; **14**. DOI:10.1371/journal.pmed.1002306.

11 Rasella D, Alves FJO, Rebouças P, *et al.* Long-term impact of a conditional cash transfer programme on maternal mortality: a nationwide analysis of Brazilian longitudinal data. *BMC Med* 2021; **19**. DOI:10.1186/s12916-021-01994-7.

12 Ramos D, da Silva NB, Ichihara MY, *et al.* Conditional cash transfer program and child mortality: A cross-sectional analysis nested within the 100 Million Brazilian Cohort. *PLoS Med* 2021; **18**. DOI:10.1371/journal.pmed.1003509.

13 Aguila E, López-Ortega M, Robledo LMG. Non-contributory pension programs and frailty of older adults: Evidence from Mexico. *PLoS One* 2018; **13**. DOI:10.1371/journal.pone.0206792.

14 Riumallo-Herl C, Aguila E. The effect of old-age pensions on health care utilization patterns and insurance uptake in Mexico. *BMJ Glob Heal* 2019; **4**. DOI:10.1136/bmjgh-

2019-001771.

15 Lindert K, Linder A, Hobbs J, de la Brière B. The Nuts and Bolts of Brazil's Bolsa Família Program: Implementing Conditional Cash Transfers in a Decentralized Context. *Soc Prot Discuss Pap Ser* 2007.

16 Hessel P, López LC, Ordóñez-Monak I, González-Uribe C. The relation between social pensions and health among poor older individuals in Colombia: A qualitative study. *Ageing Soc* 2020. DOI:10.1017/S0144686X20001361.

17 Juarez L, Pfutze T. The Effects of a noncontributory pension program on labor force participation: The case of 70 y Más in Mexico. *Econ Dev Cult Change* 2015; **63**. DOI:10.1086/681668.

18 Huang W, Zhang C. The Power of Social Pensions: Evidence from China's New Rural Pension Scheme. *Am Econ J Appl Econ* 2021; **13**: 179–205.

19 Bertranou FM, Van Ginneken W, Solorio C. The impact of tax-financed pensions on poverty reduction in Latin America: Evidence from Argentina, Brazil, Chile, Costa Rica and Uruguay. *Int Soc Secur Rev* 2004; **57**. DOI:10.1111/j.1468-246X.2004.00200.x.

20 Duflo E. Child outcomes in Africa: Child health and household resources in South Africa: Evidence from the old age pension program. *Am Econ Rev* 2000; **90**. DOI:10.1257/aer.90.2.393.

21 Duflo E. Grandmothers and granddaughters: Old-age pensions and intrahousehold allocation in South Africa. *World Bank Econ Rev* 2003; **17**. DOI:10.1093/wber/lhg013.

22 Ponczek V. Income and bargaining effects on education and health in Brazil. *J Dev Econ* 2011; **94**. DOI:10.1016/j.jdeveco.2010.01.011.

23 Hone T, Mirelman AJ, Rasella D, *et al.* Effect of economic recession and impact of health and social protection expenditures on adult mortality: a longitudinal analysis of 5565 Brazilian municipalities. *Lancet Glob Heal* 2019; **7**. DOI:10.1016/S2214-109X(19)30409-7.

24 Mariani, C. B. Gomes, E. C. Cenci, D. R. Queiroz RF de. Financiamento da Assistência Social no Brasil Nota Técnica de Monitoramento (2019). 2019.

25 Salata AR, Ribeiro MG. Boletim Desigualdade nas Metrópoles (n. 09). Observatório das Metrópoles. Porto Alegre/RS, 2022.

26 Paiva AB, Mesquita ACS, Jaccoud L, Passos L. [The new tax regime and its implications for social assistance policy in Brazil.] [Portuguese]. *Inst Pesqui Econômica Apl* 2016; **27**.

27 Vieira, F. S. Benevides RPDS. [The new tax regime and its implications for social assistance policy in Brazil.] [Portuguese]. Technical Note No.27. 2016.

28 Rossi P, Dweck E. Impacts of the New Fiscal Regime on health and education Impactos. *Cad Saude Publica* 2016; **32**.

29 Lawlor DA, Tilling K, Davey Smith G. Triangulation in aetiological epidemiology. *International Journal of Epidemiology* 2016; **45**: 1866–86.

© 2024 Aransiola TJ et al. *JAMA Network Open.*
