# Supplementary material for: Current and Projected Mortality and Hospitalization Rates Associated With Conditional Cash Transfer, Social Pension, and Primary Health Care Programs in Brazil, 2000-2030
Source: JAMA Netw Open. 2024 Apr 22;7(4):e247519. doi: 10.1001/jamanetworkopen.2024.7519 (PMC11036142; doi:10.1001/jamanetworkopen.2024.7519)
Supplement: Supplement 2. — Data Sharing Statement [file jamanetwopen-e247519-s002.pdf]

Data Sharing Statement

Aransiola. Current and Projected Mortality and Hospitalization Rates Associated With Conditional Cash Transfer, Social Pension, and Primary Health Care Programs in Brazil, 2000-2030. *JAMA Netw Open*. Published April 22, 2024. doi:10.1001/jamanetworkopen.2024.7519

Data
Data available: No
